# Isolation and genomic characterization of five novel strains of Erysipelotrichaceae from commercial pigs

**DOI:** 10.1186/s12866-021-02193-3

**Published:** 2021-04-23

**Authors:** Jinyuan Wu, Min Liu, Mengqing Zhou, Lin Wu, Hui Yang, Lusheng Huang, Congying Chen

**Affiliations:** National Key Laboratory for Swine genetic improvement and production technology, Ministry of Science and Technology of China, Jiangxi Agricultural University, NanChang, Jiangxi Province 330045 People’s Republic of China

**Keywords:** Pig, Isolation, Erysipelotrichaceae strains, Carbohydrate metabolism, PUL

## Abstract

**Background:**

Members of the Erysipelotrichaceae family have a high abundance in the intestinal tract of mammals, and have been reported to be associated with host metabolic disorders and inflammatory diseases. In our previous study, we found that the abundance of Erysipelotrichaceae strains in the cecum was associated with the concentration of N-acetylgalactosamine (GalNAc). However, only a few members of Erysipelotrichaceae have been isolated and cultured, and their main characteristics, genomic information and the functional capacity of carbohydrate metabolism remain unknown.

**Results:**

In this study, we tested 10 different kinds of commercially available media and successfully isolated five Erysipelotrichaceae strains from healthy porcine feces. The five isolates were Gram-positive, and their colonies on Gifu anaerobic medium (GAM) or modified GAM were approximately 0.25–1.0 mm in diameter, and they were circular, white, convex, moist, translucent, and contained colony margins. These isolates were subjected to Oxford Nanopore and Illumina whole-genome sequencing, genome assembly, and annotation. Based on whole-genome sequences, the five strains belong to *Erysipelotrichaceae bacterium OH741_COT-311*, *Eubacterium sp. AM28–29*, and *Faecalitalea cylindroides*. The GC content of the five strains ranged from 34.1 to 37.37%. Functional annotation based on the Kyoto encyclopedia of genes and genomes pathways revealed tens to hundreds of strain-specific proteins among different strains, and even between the strains showing high 16S rRNA gene sequence identity. Prediction analysis of carbohydrate metabolism revealed different capacities for metabolizing carbohydrate substrates among Erysipelotrichaceae strains. We identified that genes related to the GalNAc metabolism pathway were enriched in the genomes of all five isolates and 16 Erysipelotrichaceae strains downloaded from GenBank, suggesting the importance of GalNAc metabolism in Erysipelotrichaceae strains. Polysaccharide utilization loci (PUL) analysis revealed that the strains of Erysipelotrichaceae may have the ability to utilize plant polysaccharides.

**Conclusions:**

The present study not only reports the successful isolation of novel Erysipelotrichaceae strains that enrich the cultured strains of Erysipelotrichaceae, but also provided the genome information of Erysipelotrichaceae strains for further studying the function roles of Erysipelotrichaceae in host phenotypes.

**Supplementary Information:**

The online version contains supplementary material available at 10.1186/s12866-021-02193-3.

## Background

Mammalian intestines are colonized by trillions of microorganisms, most of which are bacteria. Several studies have reported that the microbial community extensively impacts host health by influencing intestinal epithelial cell proliferation, local and systemic immunity, and metabolism. Intestinal dysbacteriosis is associated with obesity, inflammatory bowel disease (IBD), nonalcoholic fatty liver disease, and metabolic syndrome [1–4]. Diet, environment, and host genetics influence the composition of the gut microbiota, and contribute to the high compositional diversity between individuals, which are comparable to unique fingerprints [5–8]. Studies on the functional capacity of the gut microbiota are urgently needed to elucidate how the microbiome interacts with the host and influences host health. However, most gut bacteria are considered to be “unculturable” in the laboratory [9]. In recent years, 16S ribosomal RNA (rRNA) gene and metagenomic sequencing techniques have been widely used in studies of the gut microbiome and have detected many bacterial species that were previously uncultured. However, the mechanism by which these uncultured bacterial species colonize and propagate in the gut, and their impact on host physiology is currently unknown. Therefore, obtaining a pure culture of these microbiota is essential to determine their roles in the gut microbiome.

Erysipelotrichaceae is a family comprising of anaerobic [10], facultative anaerobic [11] or aerobic [12] bacteria of the order Erysipelotrichales and the Firmicutes phylum [13], and was first described by Verbarg et al. [13]. Members of the Erysipelotrichaceae family have been reported to be associated with host metabolic disorders and inflammatory diseases. For example, Martinez et al. [14] observed a strong correlation between the presence of Erysipelotrichaceae and host cholesterol metabolites. Fleissner et al. [15] described an increased abundance of Erysipelotrichaceae in mice that are fed a high-fat or western diet. Kaakoush et al. [16], and Nagao-Kitamoto et al. [17] found that the gut levels of Erysipelotrichaceae change during the development of IBD in human and animal models. Moreover, the members of Erysipelotrichaceae are highly immunogenic and produce broad-spectrum antibiotics [17]. Ding et al. [18] found that the relative abundance of *Erysipelotrichi* was positively correlated with tumor necrosis factor alpha levels in chronic HIV infections. Palm et al. [19] observed that an unclassified Erysipelotrichaceae has a stronger ability to bind with immunoglobulin A than those of other members in the gut microbiota. In addition, our previous study found the host ABO genotypes can affect the abundance of Erysipelotrichaceae strains through influencing the concentration of N-acetylgalactosamine (GalNAc) in the cecum [20]. However, little information has been known about the functional capacities of Erysipelotrichaceae strains metabolizing carbohydrates. Although several strains of Erysipelotrichaceae have been isolated from the feces, oral cavity, and gastrointestinal tract of mammals [21], most of the members from Erysipelotrichaceae have not been cultured, and their genomic and functional information have not yet been elucidated [16].

To characterize the functional capacity of Erysipelotrichaceae, especially, in the metabolism of carbohydrate, we isolated five strains of Erysipelotrichaceae from fresh feces of pigs using Gifu anaerobic media (GAM) and modified GAM (mGAM) media. Next-generation sequencing was performed to study the whole-genome characteristics of these five novel strains of Erysipelotrichaceae. Genomic annotation and phylogenetic analysis revealed both the genetic diversity and conservation among the five strains. We then focused on the potential capacity of these Erysipelotrichaceae strains in the utilization of GalNAc and polysaccharides.

## Results

### Morphological characterization and phylogenetic relationships of five Erysipelotrichaceae isolates

To isolate novel members of Erysipelotrichaceae, we collected stool samples from 24 healthy pigs, and isolated Erysipelotrichaceae strains under anaerobic conditions using selective and non-selective media. A total of 10 media were chosen to cultivate a wide range of bacterial species. Most of the media were commercially available and had desirable features including an abundance of nutrients as an energy source, and fixed culturing conditions (aerobic/anaerobic, pH, and sterilizing temperature). A total of 169 isolates were re-streaked for purification, and then full-length 16S rRNA gene sequencing was performed for taxonomic annotation. The workflow for isolating Erysipelotrichaceae strains is shown in Additional file 1: Fig. S1. We successfully isolated five strains of Erysipelotrichaceae, and all of them showed growth on GAM or mGAM medium. This result suggested that GAM and mGAM sufficiently supported the growth of certain members of Erysipelotrichaceae*.* We then cultured the five isolates in aerobic condition, but none of these five strains could grow, suggesting that they are anaerobic bacteria. We observed the morphological characteristics of the colonies of the five strains. The colonies of the five strains were circular, white, convex, moist, translucent, and contained entire margins. The only difference in the morphological characteristics was the size of the colonies. The size of the colonies of the isolates 4–8-110 and 4–15-1 was approximately 0.25 mm in diameter, and the diameter of the colonies of the other three isolates was approximately 1 mm when cultured at 37 °C at pH 7.0 on GAM agar under strictly anaerobic conditions for two days.

We first used the online RDP classifier to classify the taxonomy of five isolates based on the full-length 16S rRNA gene sequences. The result showed that the 4–8-110 and 4–15-1 were most likely to belong to the genus *Bulleidia*; the isolates 4–6-57 and 5–26-39 might belong to *Faecalicoccus*; and the isolate 4–2-123 was a strain in *Holdemanella*. However, the exact taxonomies of these four isolates should need to be determined based on whole-genome sequences. And then, we constructed a phylogenetic tree of 30 strains of Erysipelotrichaceae based on the full-length 16S rRNA gene sequences of five isolates from this study and 25 strains from NCBI (Additional file 8: Table S1). The results demonstrated that the five new strains from this study were distributed in two separate clades, and were distinct from the 25 strains reported previously (Additional file 2: Fig. S2). The isolates 4–8-110 and 4–15-1 showed a high sequence identity (99.93%) to each other and formed a monophyletic group. The strains 4–2-123, 4–6-57, and 5–26-39 were placed in another clade. Compared to 4–2-123, the isolate 5–26-39 shared a higher sequence identity with 4–6-57 (4–2-123, 92.15%; 5–26-39, 100%).

### Whole-genome sequencing of the five isolates

**Fig. 1:**
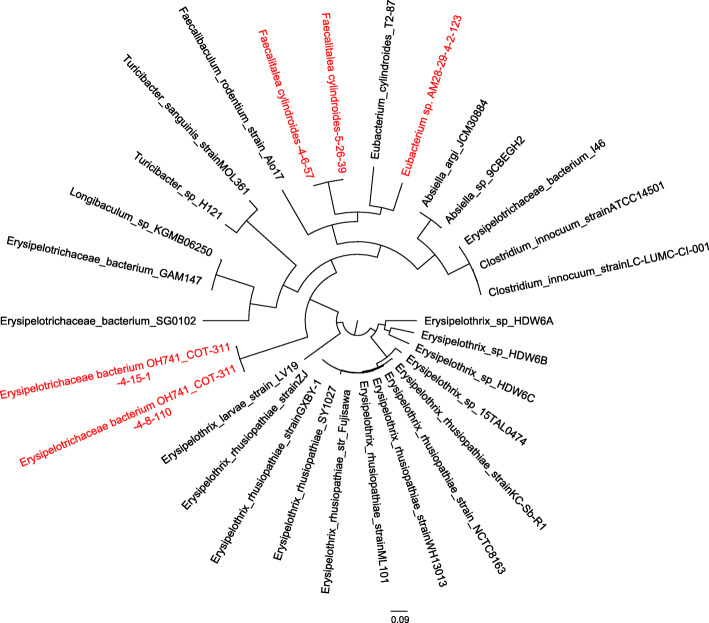
Maximum likelihood phylogenetic tree of 30 Erysipelotrichaceae strains based on whole-genome sequences. The tree shows the phylogenetic relationships of five strains isolated in this study and 25 strains downloaded from the NCBI database using OrthoFinder (v2.5.2)

To investigate the genomic characteristics of the five strains of Erysipelotrichaceae isolated in this study, all five isolates were sequenced via third-generation sequencing by using ONT. The ONT PromethION sequencing process generated a total of 10.39 Gb data, which contained 491,941 raw reads with an average length of 21.23 Kb. The sequence reads showing qscore template < 7 and reads length < 1000 bp were filtered from the raw data. Processed data containing 443,939 reads with a quality score > 9.66 (9.85 Gb) were used for further analysis (Additional file 9: Table S2). We performed de novo assembly of the strain genomes using the ONT third-generation long reads, and polished the assembly using 150-bp reads generated via second-generation sequencing. All five genome assemblies contained complete chromosomes with sizes ranging from 2.28 to 2.45 Mb, and showed 547 to 921 folds of sequencing depths. The genome sizes of the five new strains were not significantly different from that of the previously published 25 Erysipelotrichaceae strains (*t-*test, *P* = 0.22). In addition, strain 4–15-1 contained a plasmid with a genome size of 64,438 bp (Additional file 10: Table S3). To verify the integrity of the assemblies and homogeneity of sequencing, we re-mapped the clean reads to the assembled genomes, and assessed the sequencing depth by sliding the genomes using non-overlapping 1000-bp windows (Additional file 3: Fig. S3), the results showed that the sequencing depths of all five strains were sufficiently high. And we successfully constructed a total of five fully circularized single-contig genomes and a plasmid (Additional file 4: Fig. S4). Using the assembled whole-genome sequences of five isolates, we first classified the taxonomies of five isolates based on the annotation of all genes of each isolate to the RefSeq database, and found that the strain 4–8-110 and 4–15-1 were classified into *Erysipelotrichaceae bacterium OH741*, strain 4–2-123 was estimated to *Eubacterium sp. AM28–29*, and the strain 4–6-57 and 5–26-39 were sorted into *Faecalitalea cylindroides*. And then, we constructed the phylogenetic tree of five isolates and the other 25 Erysipelotrichaceae strains from NCBI GenBank using whole-genome sequences. The five isolates were distinctly distributed in three separate clades. Two *Erysipelotrichaceae bacterium OH741* strains formed a monophyletic group, two *Faecalitalea cylindroides* strains were located in a clade, and the *Eubacterium sp. AM28–29* strain was clustered with *Eubacterium cylindroides_T2–87* and distributed in another clade (Fig. 1)*.* Finally, we calculated the average nucleotide identity (ANI) values among whole-genome sequences of five isolated strains. The ANI value was 97.35% between *Erysipelotrichaceae bacterium OH741* strains (4–8-110 and 4–15-1), and 98.87% between *Faecalitalea cylindroides* strains (4–6-57 and 5–26-39). However, the ANI values were less than 75% between each other of other strains (Additional file 11: Table S4). This result further suggested that the isolates 4–8-110 and 4–15-1 should belong to the species *Erysipelotrichaceae bacterium OH741*, and the isolates 4–6-57 and 5–26-39 should be the species *Faecalitalea cylindroides*.

Prediction analysis of the five genomes identified an average of 2356 (ranging from 2281 to 2475) complete protein CDSs, and an average of 59 (ranging from 48 to 73) tRNA genes with a mean size of 4635 bp. The genomes of the strains 4–8-110, 4–6-57, and 5–26-39 contained clustered regularly interspaced short palindromic repeat (CRISPR) sequences. However, none of these five strains contained genomic islands (Additional file 12: Table S5). We then examined the compositions of the four nucleotide bases in these five genomes and found that there was no significant difference in the GC content among the five strains, which ranged from 34.1% for strain 5–26-39, to 37.37% for strain 4–8-110 (Additional file 5: Fig. S5a). Furthermore, using the shell script annotation pipeline, the putative CDSs could be annotated to six reference databases. Most of the CDSs were annotated to the Refseq and Pfam databases. However, only approximately 50% of CDSs were annotated to the KEGG [22], COG and GO databases, and even fewer CDSs could be annotated to the TIGRFAMs database (Additional file 5: Fig. S5b).

### Metabolic capacity of the five isolates of Erysipelotrichaceae

**Fig. 2:**
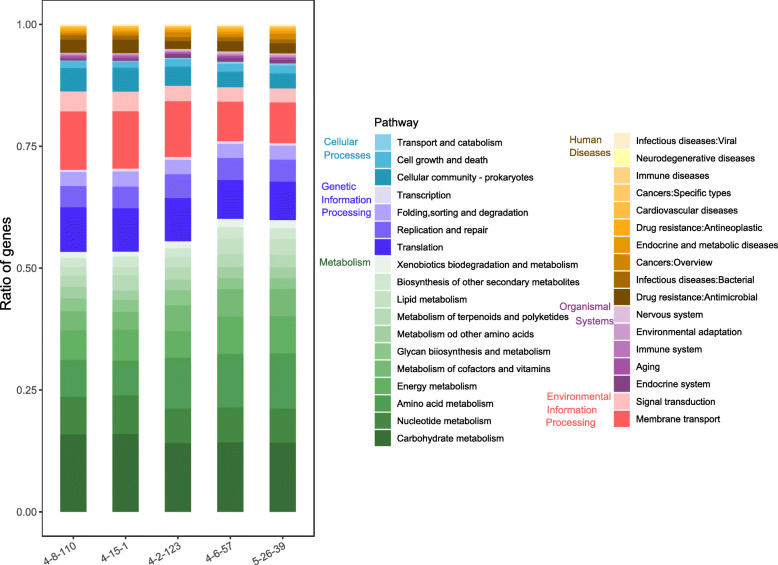
Comparison of the functional capacities of five Erysipelotrichaceae isolates based on the functional classification of all proteins (coding sequences, CDSs) by annotating them to the KEGG pathways

To predict the metabolic capacity of the five isolates, we focused on the functional classification of all CDSs by annotating them to the KEGG database. As shown in Fig. 2, we found that more than 50% of the genes were related to metabolism, such as carbohydrate, nucleotide, amino acid, and energy metabolism in all five isolates. In addition, the function terms related to genetic information processing including translation, replication and repair, and transcription; the terms associated with environmental information processing, for example, membrane transport and signal transduction; and the function terms related to cellular processes, for example cellular community - prokaryotes, were enriched by genes of the five strains. These genes account for up to 40% of the total genes in each of the five strains. In regard to pathways associated with human diseases, most genes were classified into drug resistance, antimicrobial, infectious diseases, and bacterial infections. We further analyzed the antimicrobial resistance of five Erysipelotrichaceae isolates, and only one antimicrobial resistant gene related to vancomycin resistance was found in the genomes of all the five strains. Only a few genes were annotated to organismal systems.

**Fig. 3:**
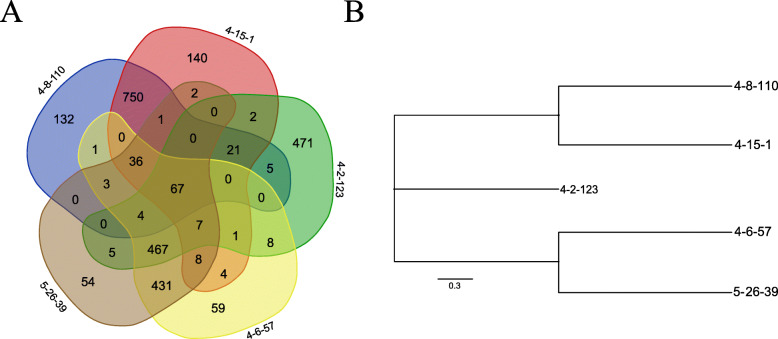
The numbers of shared and strain-specific proteins of five Erysipelotrichaceae isolates. **a** Venn diagram showing the numbers of shared and strain-specific proteins of five Erysipelotrichaceae strains. **b** The phylogenetic tree of five Erysipelotrichaceae strains constructed with the shared proteins using the Maximum Likelihood method (1000 × boostrap) and ploted using MEGA7

To further investigate the potential metabolic functions in the five Erysipelotrichaceae strains, we extracted protein sequences that could be classified into functional pathways. Using the online software Venn, we found 67 protein sequences that were shared among the five strains (Fig. 3a). In particular, although the isolates 4–8-110 and 4–15-1 showed 99.93% sequence identity of the full-length 16S rRNA gene, 132 and 140 strain-specific proteins, respectively, were identified in the two strains. Similarly, 54 and 59 strain-specific proteins were detected in isolates 5–26-39 and 4–6-57, respectively, which were clustered into one clade using full-length 16S rRNA gene sequences. To further confirm the classification of the five isolates, a phylogenetic tree was generated using these 67 protein sequences shared among the five strains (Fig. 3b). The result was quite similar to that obtained using 16S rRNA gene and whole genome sequences although the positions of the strain 4–2-123 in two phylogenetic trees show a little difference (Figs. 1 and 3b and Additional file 2: Fig. S2). We further investigated the functional classification of these 67 common proteins via alignments to the KEGG pathways. As expected, the shared proteins were mainly enriched in pathways related to fundamental metabolic processes, such as amino acid, carbohydrate, and nucleotide metabolism. The shared proteins were also enriched in the pathways associated with genetic information processing including folding, sorting and degradation, translation, replication, and repair of nucleotides. We also found that pathways related to environmental information processing, for example, ABC transporters, phosphotransferase system (PTS), and bacterial secretion system were enriched by the shared proteins (Additional file 6: Fig. S6 and Additional file 13: Table S6).

**Fig. 4:**
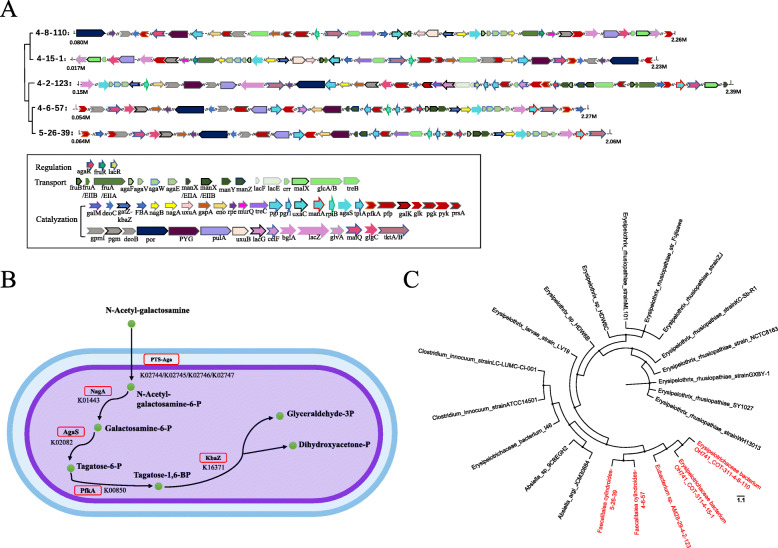
Organization and the pathway of genes related to the metabolism of carbohydrate substrates in the genome of five Erysipelotrichaceae isolates. **a** Organization of the genes related to the metabolism of 13 carbohydrate substrates in the genomes of five strains. Each arrow indicates a coding sequence (CDS) involved in the carbohydrate metabolism. All arrows were colored according to their functional roles. The detailed information about gene represented by each arrow is listed in Table S6 and the box under this Figure. “//” indicates a gap between two genes > 5 Kb. **b** The pathway for transport and catabolism of N-acetyl- galactosamine (GalNAc) in the five strains. The pathway was plotted with the relevant enzymes named by their gene symbols. **c** Phylogeny of Erysipelotrichaceae strains using the protein sequences encoded by genes in the pathway of GalNAc metabolism*.* The phylogenetic dendrogram was inferred by using the Maximum Likelihood method (1000 × replicates) and exhibited by FigTree

We subsequently focused on the pathways of carbohydrate metabolism that were enriched by shared proteins, to evaluate the functional capacity related to carbohydrate utilization. A total of seven carbohydrate metabolism pathways related to 13 carbohydrate substrates were enriched by the shared proteins (Table 1). Two *Erysipelotrichaceae bacterium OH741_COT-311* strains (4–8-110 and 4–15-1) may have the potential to utilize 11 substrates via carbohydrate metabolism according to the KEGG orthologues (KOs) of all CDSs. *Eubacterium sp. AM28–29* strain (4–2-123) may be able to use 10 out of 13 substrates. For two *Faecalitalea cylindroides* strains (4–6-57 and 5–26-39), the pathways related to the metabolism of eight substrates were enriched by shared proteins. We also observed that there were seven substrates that may be utilized by all five strains, such as GalNAc, glucose, and glycogen. Notably, lactose metabolism was only annotated to the genes of the *Eubacterium sp. AM28–29* strain (Table 1). To further elucidate the genome structure of the pathways associated with the metabolism of the 13 carbohydrate substrates in the five strains, we extracted the 61 KOs that constituted the metabolic pathways of carbohydrate metabolism, including PTS transport system, catalysis, and the regulation of related genes (Additional file 14: Table S7), and constructed the genomic structure of the pathways of carbohydrate metabolism in each of the five isolates (Fig. 4a). The arrangements of genes related to carbohydrate metabolism in the genomes of two *Erysipelotrichaceae bacterium OH741_COT-311* strains were approximately consistent with each other, although the direction of gene organization was opposite (on the opposite strand). The organization of the genes related to carbohydrate metabolism of two *Faecalitalea cylindroides* strains was also shown to be highly similar to each other, but different from that of *Eubacterium sp. AM28–29* strain. Furthermore, we evaluated the distribution and organization of carbohydrate metabolism-related genes in 14 of the 25 genomes downloaded (Additional file 7: Fig. S7). The 14 strains that were chosen represented each clade of the phylogenetic tree based on full-length 16S rRNA gene sequences. The number and organization of the carbohydrate metabolism-related genes were different among the five isolates, although a higher similarity of organization of these genes was observed between *Erysipelothrix ehusiopathiae* strain KC-Sb-R1 and *Erysipelothrix rhusiopathiae* strain GXBY-1, and between *Erysipelotheix rhusiopathiae* strain ML101 and *Erysipelothrix rhusiopathiae str. Fujisawa.*

**Table 1:** The metabolic pathways and corresponding carbohydrate substrates according to the shared protein sequences of five isolates

Carbohydrate metabolism	Metabolic substrate	4–8-110	4–15-1	4–2-123	4–6-57	5–26-39
Galactose metabolism	N-acetyl-D-galactosamine	√	√	√	√	√
	Lactose			√		
Fructose and mannose metabolism	D-Mannose			√	√	√
	D-Fructose	√	√	√		
Amino sugar and nucleotide sugar metabolism	Glc	√	√	√	√	√
	MurNAc	√	√			
Starch and sucrose metabolism	Trehalose/Maltose	√	√			
	Glycogen	√	√	√	√	√
Glycolysis / Gluconeogenesis	D-Glucose	√	√	√	√	√
	Arbutin/Salicin	√	√	√	√	√
Pentose and glucuronate interconversions	D-Glucuronate	√	√			
Pentose phosphate pathway	β-D-Glucose-6P	√	√	√	√	√
	2-Deoxy-D-ribose-1P	√	√	√	√	√

Note: “√” means that the genome of the strains contains the carbohydrate metabolism pathway. 4–8-110 and 4–15-1: Erysipelotrichaceae bacterium OH741_COT-311 strains; 4–2-123: Eubacterium sp. AM28–29 strain, and 4–6-57 and 5–26-39: Faecalitalea cylindroides strains; *Glc* glucose, *MurNAc* N-acetylmuramate

### Import and catabolic pathway genes for GalNAc metabolism in the genomes of Erysipelotrichaceae isolates

To investigate the utilization of GalNAc in the five strains of Erysipelotrichaceae, we explored the potential import and catabolic pathways of GalNAc based on the whole genomes of the five isolates. According to studies on *Escherichia coli* [23], *Streptomyces coelicolor* [24], *Bacillus subtilis* [25, 26] and Proteobacteria [27], the import and catabolic pathways of GalNAc metabolism include two key components: (i) GalNAc transporter systems (AgaPTS: *agaF*, *agaV*, *agaW*, *agaE*) which transport GalNAc into bacterial cells across the membrane; (ii) catabolic enzymes that convert GalNAc into intermediates, including GalNac-6P deacetylases, *nagA*; GalN-6P isomerase, *agaS*; tagatose-6P kinase, *pfkA*, and tagatose-1,6-BP aldolase, *gatZ-kbaZ*. We found all of the above listed genes in the genomes of the five isolates (Fig. 4b). We further investigated whether the genomes of the 25 Erysipelotrichaceae strains that were downloaded from the NCBI database as described above also contained the genes associated with GalNAc metabolism. We used integrative approaches by combining the prodigal software and online KEGG annotation. Results showed that 16 out of the 25 Erysipelotrichaceae strains contained GalNAc metabolism-associated genes in their genomes (Additional file 8: Table S1). We then performed a phylogenetic relationship analysis on these GalNAc metabolism-associated genes to evaluate the diversity of GalNAc metabolism pathways in all 21 strains (16 strains from NCBI and the five isolates). The 21 strains were distributed in different clades of the phylogenetic tree and the evolutionary distance was large between each other, although all of them were associated with the GalNAc metabolism pathway (Fig. 4c).

### Prediction of polysaccharide utilization loci (PUL) in the genomes of the Erysipelotrichaceae strains

We predicted the PUL present in the genomes of 30 strains of Erysipelotrichaceae based on 75% sequence identity using dbCAN-PUL tools (Additional file 15: Table S8). The number of predicted PUL in the 30 strains ranged from 0 to 10, with a median of 3. The highest number of PUL was found to be shown by 4–15-1 (10), followed by 4–8-110 (6). The strains *Clostridium innocuum* strain ATCC14501 and *Erysipelothrix larvae* strain LV19 did not show any predicted PUL. From the predicted PUL, we found that these Erysipelotrichaceae strains may be able to use dietary plant glycans and a few polysaccharides from animal sources. Most of these strains were predicted to degrade carboxymethylcellulose, xylan, beta-glucan, and lichenan, and more than half of these 30 strains may have the potential to catabolize glycosaminoglycan, unsaturated hyaluronate disaccharide, chondroitin disaccharide, N-glycan, and pectin. Only the strain 4–15-1 may have the potential capacity for polysaccharide biosynthesis, such as capsule polysaccharide, O-antigen, and exopolysaccharide.

## Discussion

The roles of the gut microbiome in host health are difficult to characterize because many bacteria have not been cultured at present [28–30]. To solve this problem, many approaches for improving the cultivation of bacteria including various kinds of samples, media, and culture conditions have been employed to isolate unculturable bacteria [31–33]. In this study, we successfully cultured and cultured five novel strains of Erysipelotrichaceae using 10 different commercially available media. The genome structures of these five isolates were elucidated via ONT third-generation sequencing and polished via deep second-generation sequencing. We systematically predicted the functional capacities of the Erysipelotrichaceae strains in the metabolism of carbohydrates by combining the genomic information of 25 previously characterized Erysipelotrichaceae strains downloaded from the NCBI. We also analyzed the functions of the Erysipelotrichaceae strains in the metabolism of GalNAc and polysaccharides. The present study not only characterizes the strains of Erysipelotrichaceae for culture-based studies on the activities of Erysipelotrichaceae, but also provides knowledge on Erysipelotrichaceae genomes that facilitates the study of the relationships between Erysipelotrichaceae strains and host traits. All five isolates could be cultured on media of GAM and mGAM containing serum and liver extracts, which were different from the other eight media. We estimated that the growth of Erysipelotrichaceae strains may require a large amount of protein, lipid, and certain growth factors, organics, vitamins, and unknown ingredients.

Full-length 16S rRNA gene sequencing analysis demonstrated that 4–8-110 showed 99.93% sequence identity with 4–15-1, and 100% sequence identity was observed between 5 and 26-39 and 4–6-57. However, significant differences in gene content were found between these two pairs of strains. The genome size of 4–15-1 was larger than that of 4–8-110, and the genome sizes of 4–6-57 and 5–26-39 were also different. Furthermore, these strains showed different numbers of CDSs, and the taxonomy annotations of five strains were also different based on 16S rRNA genes and whole-genomes. These results suggested the limitation in the analysis of the strain-level diversity using 16S rRNA gene sequencing. However, the analyses based on whole-genome sequences could clearly indicate that the isolates 4–8-110 and 4–15-1, and the isolates 4–6-57 and 5–26-39 belong to a species separately. This different genetic contents between strains were also reported in other bacteria, For example, previous studies have reported that the presence of distinct *Prevotella copri* strains in the gut metagenomes associated with various dietary habits, and the pangenomes of *P. copri* found that the different *P. copri* strains show distinct gene repertoires and a strain-level diversity [34, 35].

Several bacterial species in the gut are involved in the fermentation and catabolism of dietary fibers, including polysaccharides, thus generating absorbable micromolecules such as short-chain fatty acids (SCFAs), which are primarily produced in the cecum and colon and play key roles in regulating host metabolism, immune system, and cell proliferation [36, 37]. According to previous studies, the levels of intestinal Erysipelotrichaceae are positively correlated with carbohydrate consumption [38]. Similarly, the abundance of Erysipelotrichaceae has a positive association with SCFAs levels [39]. Moreover, Nilsson et al. found that members of Erysipelotrichaceae may produce SCFAs [40]. By performing prediction analysis of PUL in this study, we found that the five isolates of Erysipelotrichaceae may also have the capacity to metabolize plant polysaccharides such as xylan and lichenan. Compared to *P. copri* [41], relatively fewer PUL were found in the genomes of Erysipelotrichaceae strains. This could be due to the fact that the dbCAN-PUL database did not contain data on Erysipelotrichaceae. Furthermore, the functional capacity for metabolizing polysaccharides needs to be confirmed via fermentation experiments.

GalNAc is present in lipopolysaccharides, which are common components of the bacterial cell wall [42, 43]. It is also linked to the carbohydrate chains of human mucins [44]. In addition, amino sugars are present in the carbohydrate chains of glycosylated proteins in both prokaryotes and eukaryotes [45, 46]. Yang et al. [20] reported that host ABO genotypes cause the different concentrations of porcine cecum lumen that further influences the abundance of several Erysipelotrichaceae strains in the porcine cecum lumen, suggesting that GalNAc as a carbohydrate source plays an important role in the growth of Erysipelotrichaceae strains.

## Conclusions

In summary, we successfully isolated and cultured five strains of Erysipelotrichaceae, and elucidated their genomic characterization in detail. We predicted the functional capacity of these five isolates in the metabolism of carbohydrates, especially in the metabolism of GalNAc and polysaccharides. These results not only characterize the novel strains of Erysipelotrichaceae but also provide basic knowledge for further studies on the functional roles of Erysipelotrichaceae in host phenotypes. However, all these functional capacities were predicted from the genomes of these Erysipelotrichaceae strains. Further metabolism experiments need to be carried out to confirm their functional capacities.

## Methods

### Isolation and culturing of bacterial strains

Fecal samples used for the isolation of Erysipelotrichaceae strains were collected from 24 pigs (age, 120 days); the pigs were fed a complete formula feed. Fresh samples were immediately transferred into an anaerobic glovebox (Electrotek, UK), which was filled with 80% nitrogen, 10% hydrogen, and 10% carbon dioxide, and then suspended in sterilized phosphate-buffered saline (PBS) (gibco, USA). The suspension was serially diluted to 10^− 6^, 10^− 7^, 10^− 8^, and 10^− 9^ with sterilized 1× PBS (pH 7.0), followed by inoculation on 10 different media including GAM (Nissui Pharmaceutical, Japan), mGAM (Nissui Pharmaceutical), Columbia agar (ELITE-MEDIA, China) containing 5% sheep blood, American type culture collection (ATCC) medium 2107 (ELITE-MEDIA), PYG medium (Hopebio, China), reinforced clostridial medium (Oxoid, UK), brain heart infusion medium (BD Biosciences, Franklin Lakes, NJ, USA), M2GSC medium, ATCC medium 27,768 (BD Biosciences) containing 5% sheep blood, and Wilkins-Chalgren anaerobe agar (Oxoid, UK). After anaerobic incubation at 37 °C for 2–5 days, the colonies were picked and streaked on the corresponding medium plates until pure colonies were obtained. Isolated colonies from the spread plates were inoculated in the corresponding broths, and then stored at − 80 °C in the broths containing 20% of glycerol (XILONG SCIENTIFIC, China). The procedures for Gram stain including primary stain, mordant, decolorization and counterstain were performed to determine Gram positive or Gram negative of the isolates.

### Full-length 16S rRNA gene sequencing

The identification of each isolate was performed via polymerase chain reaction (PCR) amplification and sequencing. The full-length 16S rRNA gene was amplified using the forward primer 27F: 5′-AGAGTTTGATCCTGGCCTCAG-3′ and reverse primer 1492R: 5′-GGTTACCTTGTTACGACTT-3’ [47]. PCR amplification was carried out under the following conditions: 96 °C for 3 min, 35 × (96 °C for 30 s, 58 °C for 30 s, 72 °C for 1 min), and 72 °C for 10 min. The purified PCR products were subjected to Sanger sequencing. Full-length 16S rRNA gene sequences were used for taxonomic classifications at the genus level based on the Ribosomal Database Project (RDP) reference database using online RDP classifier [48], and the nucleotide basic local alignment search tool (BLASTn) [49] was used to determine whether the isolates were characterized species or candidate novel species based on 97% sequence similarity with the reference genomes of bacterial species.

### Whole-genome sequencing and annotation

Five strains of Erysipelotrichaceae (named 4–8-110, 4–15-1, 4–2-123, 4–6-57, and 5–26-39) isolated in this study were selected for further whole-genome sequencing. Briefly, five strains were recovered from GAM broth. The cells were harvested at the exponential growth phase. Bacterial genomic DNA was extracted using the Blood & Cell Culture DNA Midi Kit (Qiagen, Germany) according to the manufacturer’s instructions. The integrity of the DNA samples was checked using 0.8% agarose gel electrophoresis, and the purity and quantity were measured using a NanoDrop™ One UV-Vis spectrophotometer (Thermo Fisher Scientific, USA) and a Qubit® 3.0 Fluorometer (Invitrogen, USA). The sequencing libraries were prepared using 1 μg DNA from each strain, and barcodes were added using the NBD103 and NBD114 kits by following the protocols of Oxford Nanopore Technology (ONT) [50]. The libraries were loaded onto a flow cell for real-time single-molecule sequencing using a PromethION platform (Oxford Nanopore Technologies, UK) under standard conditions. To correct the sequence errors of third-generation sequencing, a library for second-generation sequencing was prepared for each strain and sequenced using a BGISEQ platform by using a 100 bp paired-end strategy. The de novo assembly of the third-generation sequencing data was conducted using flye v2.6 (parameter: --nano-raw) [51] after performing quality control. Error correction with short reads was further performed by using pilon v1.23 (parameter: default) [51–53]. The sequencing depth of each replicon was estimated using minimap2 [54]. The coverage at each base of assembly was identified using samtools v1.3 (parameter: default) [55], and the average sequencing depth of each window was calculated using a 1000-bp window. The complete coding sequences (CDSs) were extracted from the genomes using prodigal v2.6.3 (parameter: -p None -g 11) [56], and the complete coding sequences (CDSs) were extracted. The extracted protein sequences were annotated to TIGRFAMS, Pfam, and gene ontology (GO) databases [57–59], using Interproscan (v5.25–64.0) with the following parameters: -appl Pfam, TIGRFAM, SMART – iprlookup -goterms -t p -f TSV. The protein sequences were also annotated to the Kyoto Encyclopedia of genes and genomes (KEGG) and refseq databases [60, 61] by using protein BLAST with the following parameters: -evalue 1e-05 -outfmt ‘6 std. qlen slen stitle’ -max_target_seqs 5; and annotated to the clusters of orthologous groups database [62] using rpsblast with the following parameters: -evalue 0.01 -seg no -outfmt 5. To annotate the taxonomic species of the five strains, all genes of each strain were annotated into the RefSeq database, and then the species with the most number of annotated genes was assigned to that strain. The annotation results of the different databases were visualized using ggplot in R package (v 4.0.0).

### Phylogenetic analysis and prediction of PUL

To construct the phylogenetic tree of the five isolates along with other strains of Erysipelotrichaceae reported previously, the whole-genome sequences of 25 strains of Erysipelotrichaceae were retrieved from the National Center of Biotechnology Information database (NCBI; NIH, Bethesda, MD, USA) and the accession numbers were listed in Additional file 8: Table S1. The full-length 16S rRNA gene sequences of these 25 strains were extracted from their whole-genome sequences using the 27F and 1492R primers with BLAST and samtools (v1.7).

Phylogenetic analysis was performed using the Molecular Evolutionary Genetics Analysis 7 (MEGA 7.0) software [63] after performing multiple alignments by using ClustalW [64, 65] based on the full-length 16S rRNA gene sequences or protein sequences common to all strains. Phylogenetic trees were constructed using the maximum likelihood method [66]. Evolutionary distances were calculated using the Tamura-Nei model [67]. In addition, bootstrap analysis of 1000 replicates was performed to evaluate the statistical reliability of the trees [68]. The phylogenetic tree was also constructed based on the whole-genome sequences of 30 strains using OrthoFinder (v2.5.2) under default parameters [69]. These phylogenetic trees were visualized using the FigTree software v1.4.4 (http://tree.bio.ed.ac.uk/software/figtree/). The online ANI Calculator [70] (https://www.ezbiocloud.net/tools/ani) was used to calculate the ANI values among the whole-genome sequences of five strains. The Venn (https://bioinformatics.psb.ugent.be/webtools/Venn/) were used to analyze the proteins shared among the five isolates.

The whole-genome sequences of 30 strains of Erysipelotrichaceae were used to predict the PUL using dbCAN-PUL tools (http://bcb.unl.edu/dbCAN_PUL/home) [71, 72]. The dbCAN-PUL database contains most of the experimentally verified PUL from 10 different phyla and 173 bacterial species, and comprises different metabolic systems. The predicted PUL with 75% identity were selected for further analysis.

## Supplementary Information

**Additional file 1: Figure S1.** A workflow for isolating and culturing intestinal bacterial strains.

**Additional file 2: Figure S2.** Maximum likelihood phylogenetic tree of 30 Erysipelotrichaceae strains based on full-length 16S rRNA gene sequences. The tree shows the phylogenetic relationships of five strains isolated in this study and 25 strains downloaded from the NCBI database. The clades corresponding to partitions reproduced in less than 50% bootstrap replicates are collapsed; all positions containing gaps and missing data were eliminated. NCBI, national center for biotechnology information.

**Additional file 3: Figure S3.** The distribution of sequencing depths of five isolate genomes based on non-overlapping 1000-bp windows.

**Additional file 4: Figure S4.** Circos diagrams of closed and circular genomes of the five isolates.

**Additional file 5: Figure S5.** The statistics of bases and functional composition for the genomes of five isolates.

**Additional file 6: Figure S6.** The relative KEGG pathways of the shared proteins.

**Additional file 7: Figure S7.** The organization of the genes related to the metabolisms of 13 carbohydrate substrates in the genomes of 14 Erysipelotrichaceae strains downloaded from the NCBI database.

**Additional file 8: Table S1.** Accession numbers, genome coverage and size, and the pathway of GalNAc of the strains downloaded from NCBI.

**Additional file 9: Table S2.** Statistic description for sequencing data of five isolates.

**Additional file 10: Table S3.** Genome size, the number of contigs and sequencing depth for each strain.

**Additional file 11: Table S4.** The ANI values between the five strains based on whole genomes.

**Additional file 12: Table S5.** Genome structures predicted for five isolates.

**Additional file 13: Table S6.** Annotation of proteins shared by five isolates with KEGG pathways at the different levels.

**Additional file 14: Table S7.** KEGG orthologues involving the transport (PTS system), catalyzation and regulation of the metabolisms of 13 carbohydrate substrates.

**Additional file 15: Table S8.** Prediction of PUL in the genomes of Erysipelotrichaceae strains.

## Abbreviations

16S rRNA: 16S ribosomal RNA
GAM: Gifu anaerobic medium
mGAM: Modified GAM
GalNAc: N-acetyl-galactosamine
PUL: Polysaccharide utilization loci
KOs: KEGG orthologues
SCFAs: Short-chain fatty acids
