# Supplementary material for: Isolation and genomic characterization of five novel strains of Erysipelotrichaceae from commercial pigs
Source: BMC Microbiol. 2021 Apr 23;21:125. doi: 10.1186/s12866-021-02193-3 (PMC8063399; doi:10.1186/s12866-021-02193-3)
Supplement: Supplementary file 1 — Additional file 1: Figure S1. A workflow for isolating and culturing intestinal bacterial strains. [file 12866_2021_2193_MOESM1_ESM.docx]

**Supplementary Materials**

**Isolation and genomic characterization of five novel strains of Erysipelotrichaceae from commercial pigs**

**Jinyuan Wu^1^*, Min Liu^1^,** **Mengqing Zhou^1^, Lin Wu^1^, Hui Yang^1^, Lusheng Huang^1^, Congying Chen^1^***

^1^National Key Laboratory for Swine genetic improvement and production technology, Ministry of Science and Technology of China, Jiangxi Agricultural University, NanChang, Jiangxi Province, 330045, PR China.

***Correspondence author:**

Congying Chen: [chcy75@hotmail.com](mailto:chcy75@hotmail.com);

Jinyuan Wu: [sunny940719@outlook.com](mailto:sunny940719@outlook.com).

**Supplementary Figures**


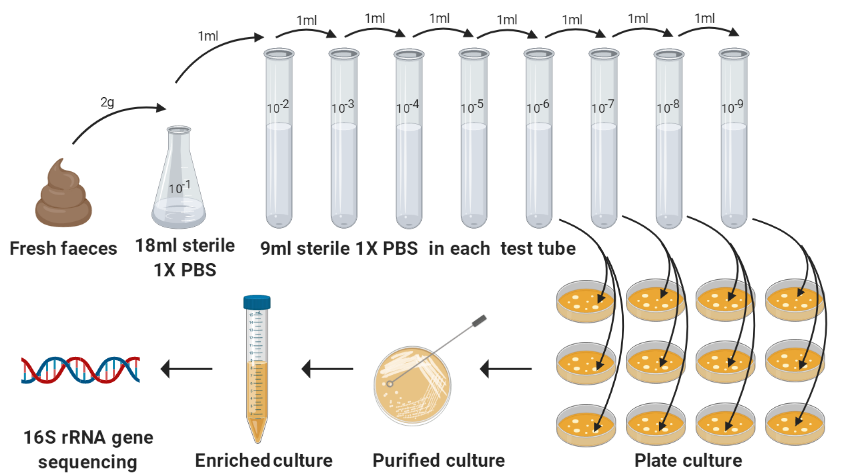


**Supplementary Figure 1.** A workflow for isolating and culturing intestinal bacterial strains. The fresh feces samples were homogenized with sterile 1x PBS and then serially diluted at 10^-6^, 10^-7^, 10^-8^ and 10^-9^. The suspensions were spread on media and each dilution was repeated three times. Plates were incubated at 37 ℃ for 3 days in an anaerobic glovebox and single colony was selected to streak and purity. Full-length 16S rRNA gene was amplified and sequenced to identify the strains. Each unique and desired isolate was frozen for further analysis. Illustrations were created with BioRender (<https://biorender.com/>).





**Supplementary Figure 2:** Maximum likelihood phylogenetic tree of 30 Erysipelotrichaceae strains based on full-length 16S rRNA gene sequences. The tree shows the phylogenetic relationships of five strains isolated in this study and 25 strains downloaded from the NCBI database. The clades corresponding to partitions reproduced in less than 50% bootstrap replicates are collapsed; all positions containing gaps and missing data were eliminated. NCBI, national center for biotechnology information.





**Supplementary Figure 3.** The distribution of sequencing depths of five isolate genomes based on non-overlapping 1,000-bp windows.


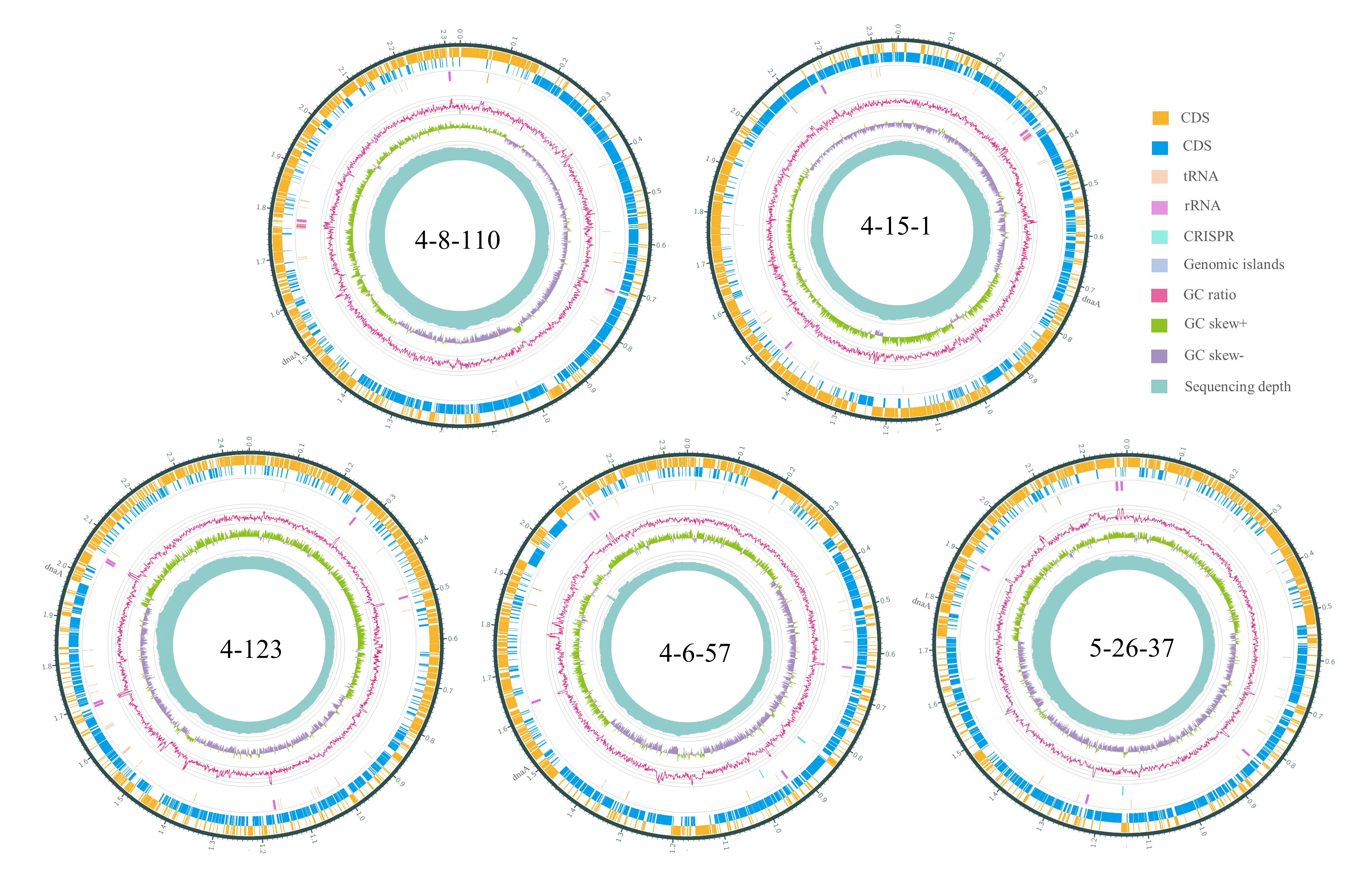


**Supplementary Figure 4.** Circos diagrams of closed and circular genomes of the five isolates. The circles from outside to inside represent coding genes in positive strand (yellow), coding genes in negative strand (blue), noncoding genes (tRNA (orange), rRNA (purple), CRISPR (aqua) and genomic islands (grey)), GC ratio (pink), GC-SKEW (green), and sequencing depth. The origin of replication is marked for each strain (dnaA). The circle maps of the genomes were visualized using the software circos (v 5.16). CRISPR, clustered regularly interspaced short palindromic repeats.


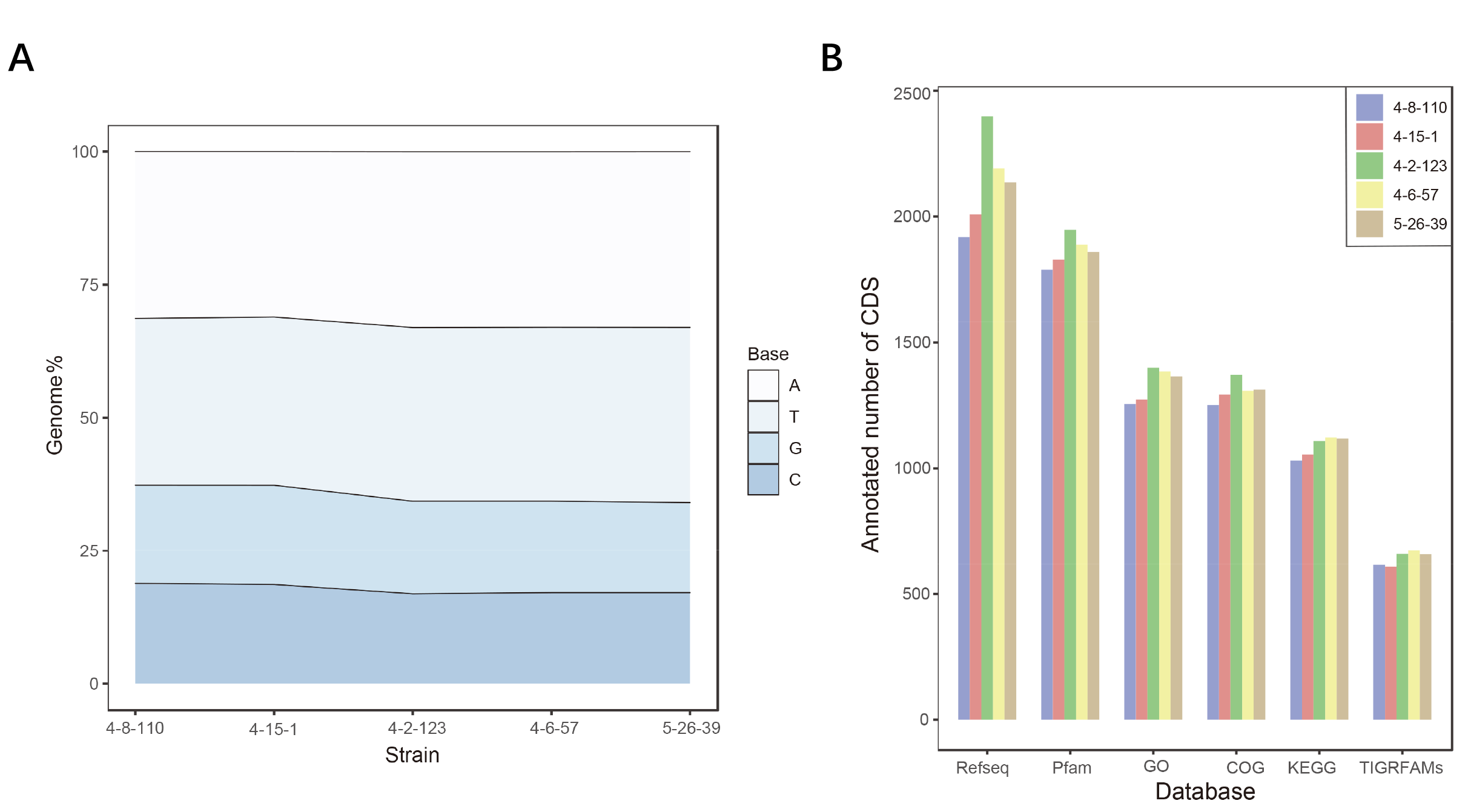


**Supplementary Figure 5.** The statistics of bases and functional composition for the genomes of five isolates. **(A)** The distibution of four bases in each genome. **(B)** Functional annotation of complete CDS in each isolates using Refseq, Pfam, GO, COG, KEGG and TIGRFAMs reference databases. The plots was obtained with “ggplot” in R package.

**
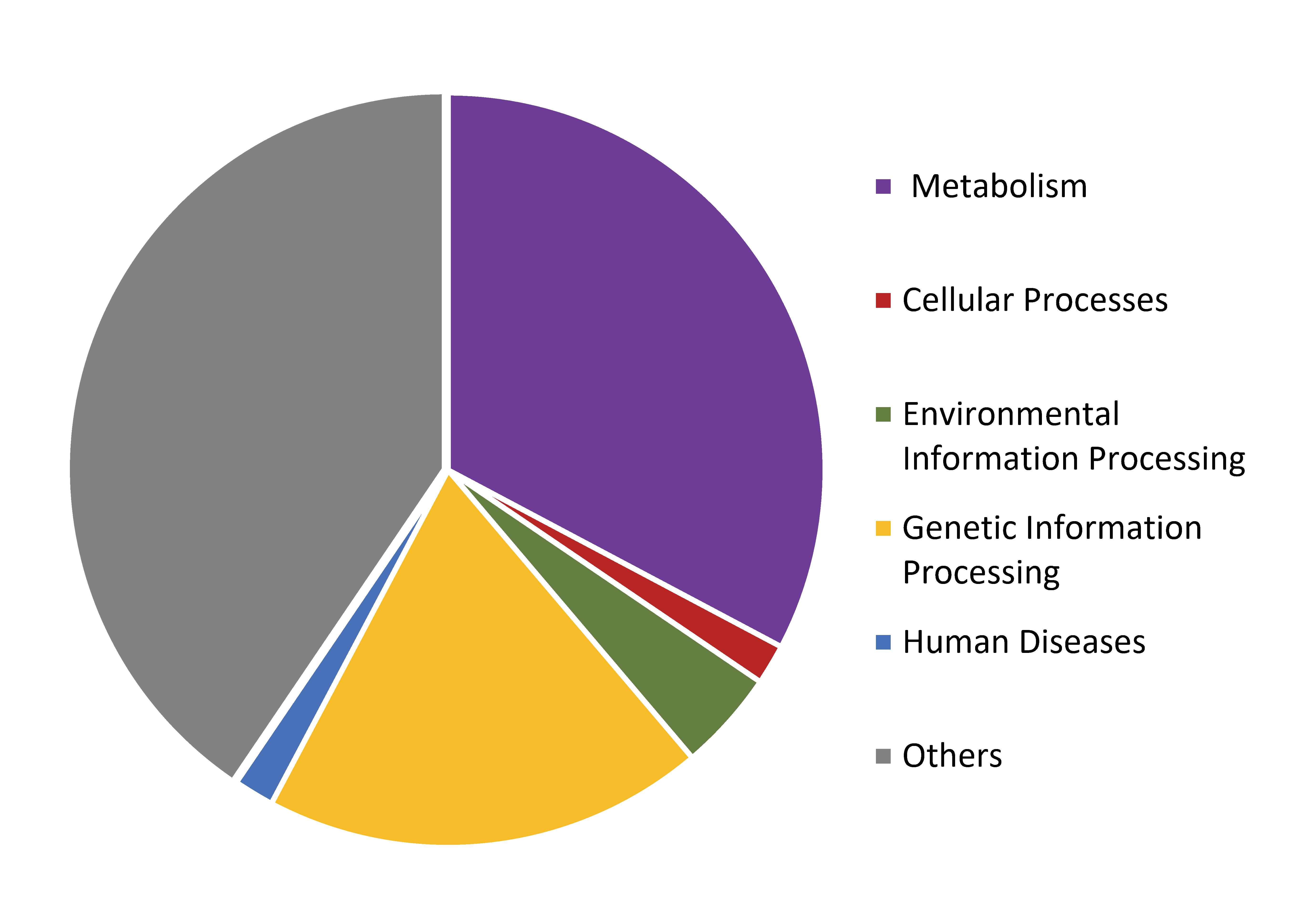
**

**Supplementary Figure 6.** Functional classification of the shared proteins based on the KEGG pathways.


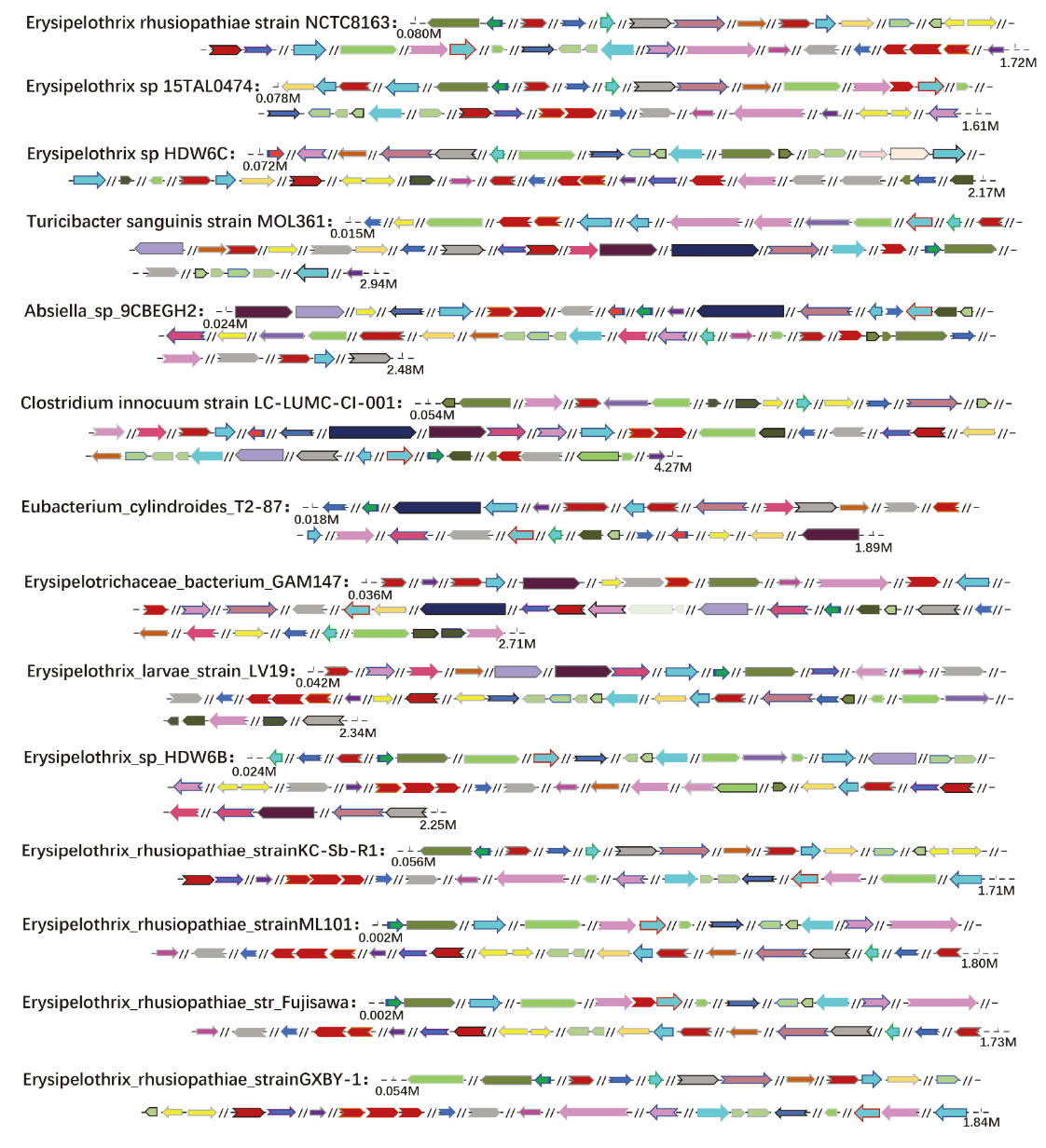


**Supplementary Figure 7.** The organization of the genes related to the metabolisms of 13 carbohydrate substrates in the genomes of 14 Erysipelotrichaceae strains downloaded from the NCBI database. Each colored arrow represents a gene involving in the metabolism of carbohydrate substrates. The detailed information about genes represented by each arrows is displayed in Table S6.
