# Supplementary material for: Isolation and genomic characterization of five novel strains of Erysipelotrichaceae from commercial pigs
Source: BMC Microbiol. 2021 Apr 23;21:125. doi: 10.1186/s12866-021-02193-3 (PMC8063399; doi:10.1186/s12866-021-02193-3)
Supplement: Supplementary file 2 — Additional file 2: Figure S2. Maximum likelihood phylogenetic tree of 30 Erysipelotrichaceae strains based on full-length 16S rRNA gene sequences. The tree shows the phylogenetic relationships of five strains isolated in this study and 25 strains downloaded from the NCBI database. The clades corresponding to partitions reproduced in less than 50% bootstrap replicates are collapsed; all positions containing gaps and missing data were eliminated. NCBI, national center for biotechnology information. [file 12866_2021_2193_MOESM2_ESM.docx]

**Supplementary Figure 2:** Maximum likelihood phylogenetic tree of 30 Erysipelotrichaceae strains based on full-length 16S rRNA gene sequences. The tree shows the phylogenetic relationships of five strains isolated in this study and 25 strains downloaded from the NCBI database. The clades corresponding to partitions reproduced in less than 50% bootstrap replicates are collapsed; all positions containing gaps and missing data were eliminated. NCBI, national center for biotechnology information.
