# Supplementary material for: Isolation and genomic characterization of five novel strains of Erysipelotrichaceae from commercial pigs
Source: BMC Microbiol. 2021 Apr 23;21:125. doi: 10.1186/s12866-021-02193-3 (PMC8063399; doi:10.1186/s12866-021-02193-3)
Supplement: Supplementary file 3 — Additional file 3: Figure S3. The distribution of sequencing depths of five isolate genomes based on non-overlapping 1000-bp windows. [file 12866_2021_2193_MOESM3_ESM.docx]

**Supplementary Figure 3.** The distribution of sequencing depths of five isolate genomes based on non-overlapping 1,000-bp windows.
