# Supplementary material for: Isolation and genomic characterization of five novel strains of Erysipelotrichaceae from commercial pigs
Source: BMC Microbiol. 2021 Apr 23;21:125. doi: 10.1186/s12866-021-02193-3 (PMC8063399; doi:10.1186/s12866-021-02193-3)
Supplement: Supplementary file 4 — Additional file 4: Figure S4. Circos diagrams of closed and circular genomes of the five isolates. [file 12866_2021_2193_MOESM4_ESM.docx]

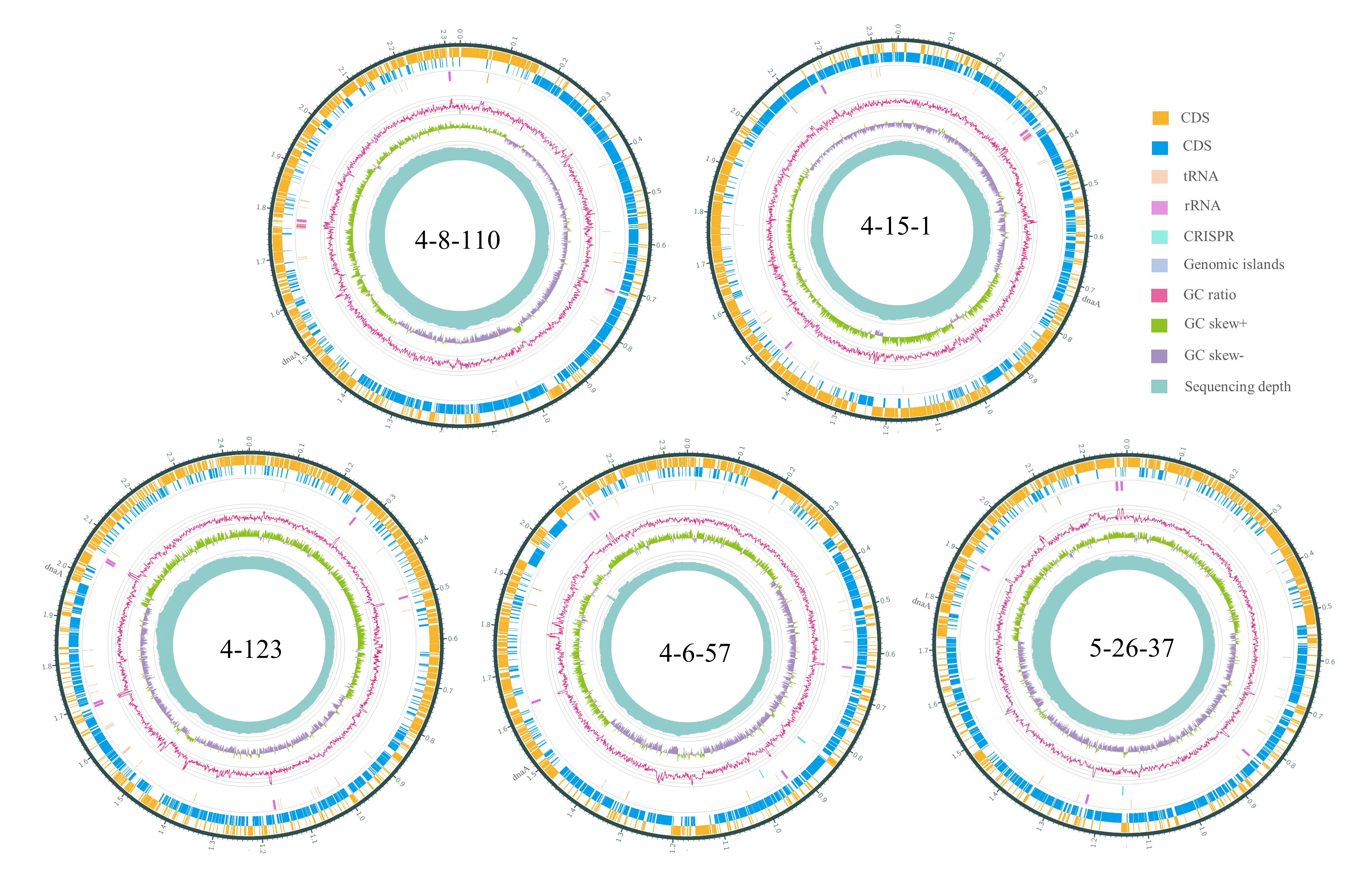


**Supplementary Figure 4.** Circos diagrams of closed and circular genomes of the five isolates. The circles from outside to inside represent coding genes in positive strand (yellow), coding genes in negative strand (blue), noncoding genes (tRNA (orange), rRNA (purple), CRISPR (aqua) and genomic islands (grey)), GC ratio (pink), GC-SKEW (green), and sequencing depth. The origin of replication is marked for each strain (dnaA). The circle maps of the genomes were visualized using the software circos (v 5.16). CRISPR, clustered regularly interspaced short palindromic repeats.
