# Supplementary material for: Isolation and genomic characterization of five novel strains of Erysipelotrichaceae from commercial pigs
Source: BMC Microbiol. 2021 Apr 23;21:125. doi: 10.1186/s12866-021-02193-3 (PMC8063399; doi:10.1186/s12866-021-02193-3)
Supplement: Supplementary file 5 — Additional file 5: Figure S5. The statistics of bases and functional composition for the genomes of five isolates. [file 12866_2021_2193_MOESM5_ESM.docx]

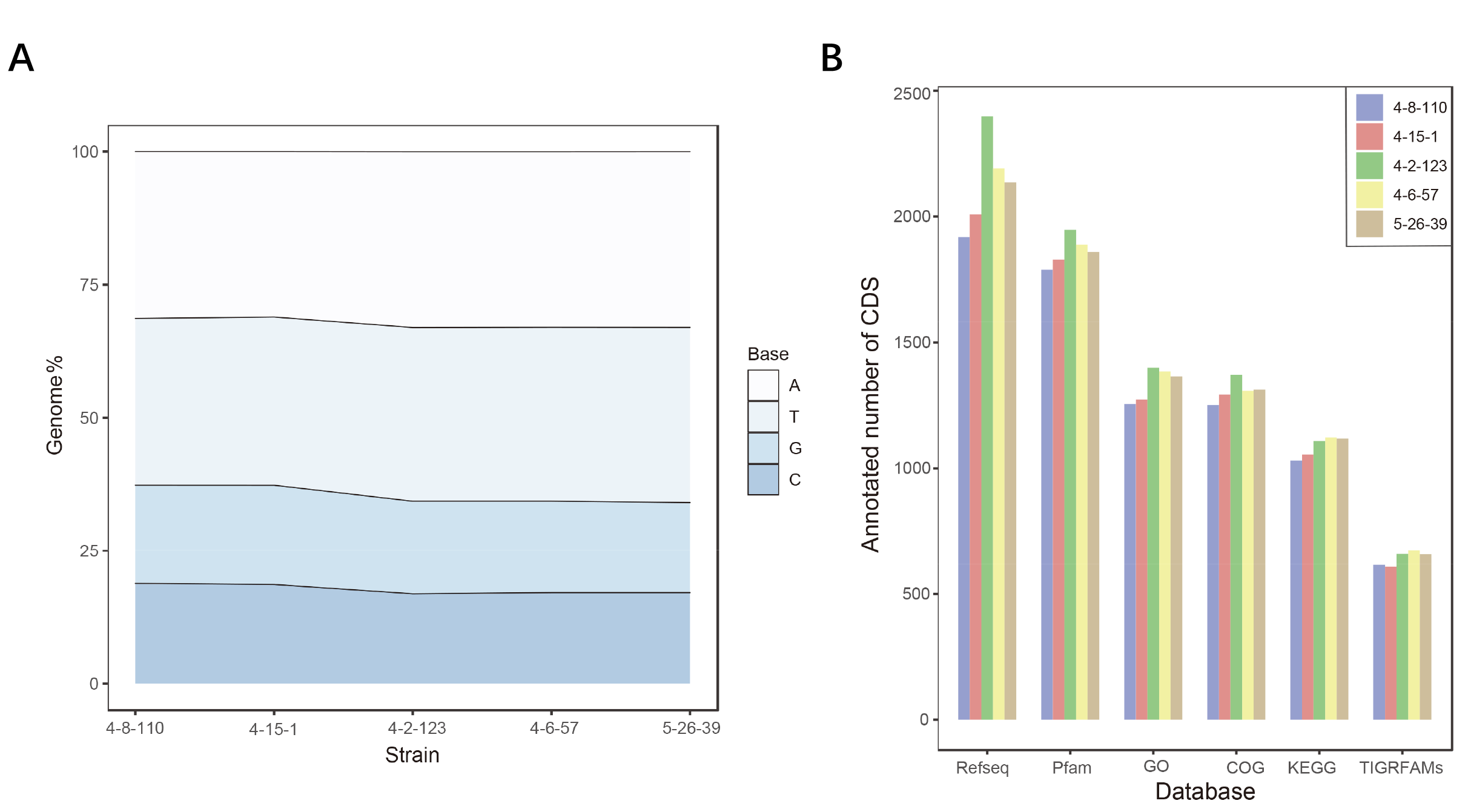


**Supplementary Figure 5.** The statistics of bases and functional composition for the genomes of five isolates. **(A)** The distibution of four bases in each genome. **(B)** Functional annotation of complete CDS in each isolates using Refseq, Pfam, GO, COG, KEGG and TIGRFAMs reference databases. The plots was obtained with “ggplot” in R package.
