# Supplementary material for: Isolation and genomic characterization of five novel strains of Erysipelotrichaceae from commercial pigs
Source: BMC Microbiol. 2021 Apr 23;21:125. doi: 10.1186/s12866-021-02193-3 (PMC8063399; doi:10.1186/s12866-021-02193-3)
Supplement: Supplementary file 6 — Additional file 6: Figure S6. The relative KEGG pathways of the shared proteins. [file 12866_2021_2193_MOESM6_ESM.docx]

**
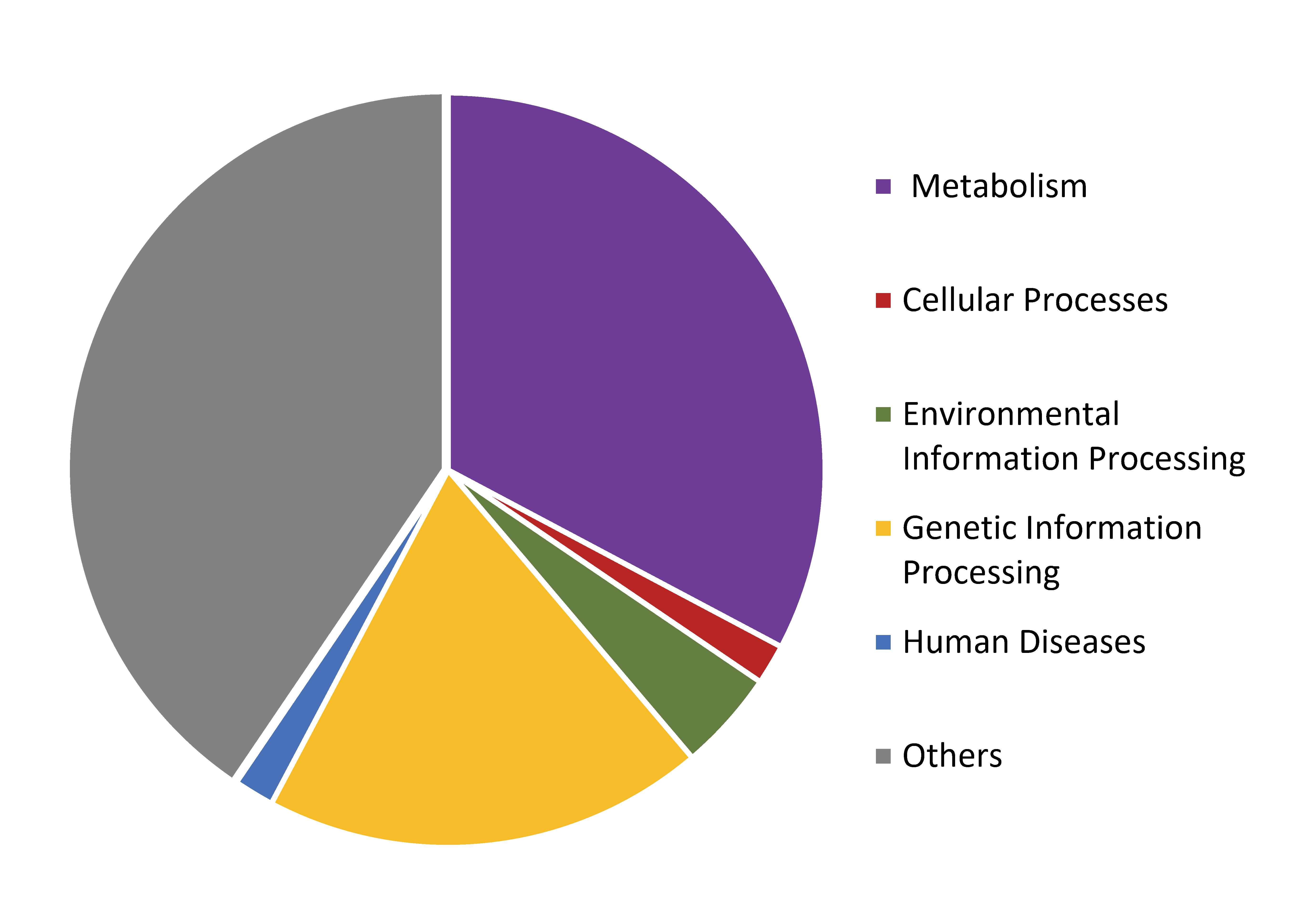
**

**Supplementary Figure 6.** Functional classification of the shared proteins based on the KEGG pathways.
