# Supplementary material for: Isolation and genomic characterization of five novel strains of Erysipelotrichaceae from commercial pigs
Source: BMC Microbiol. 2021 Apr 23;21:125. doi: 10.1186/s12866-021-02193-3 (PMC8063399; doi:10.1186/s12866-021-02193-3)
Supplement: Supplementary file 7 — Additional file 7: Figure S7. The organization of the genes related to the metabolisms of 13 carbohydrate substrates in the genomes of 14 Erysipelotrichaceae strains downloaded from the NCBI database. [file 12866_2021_2193_MOESM7_ESM.docx]

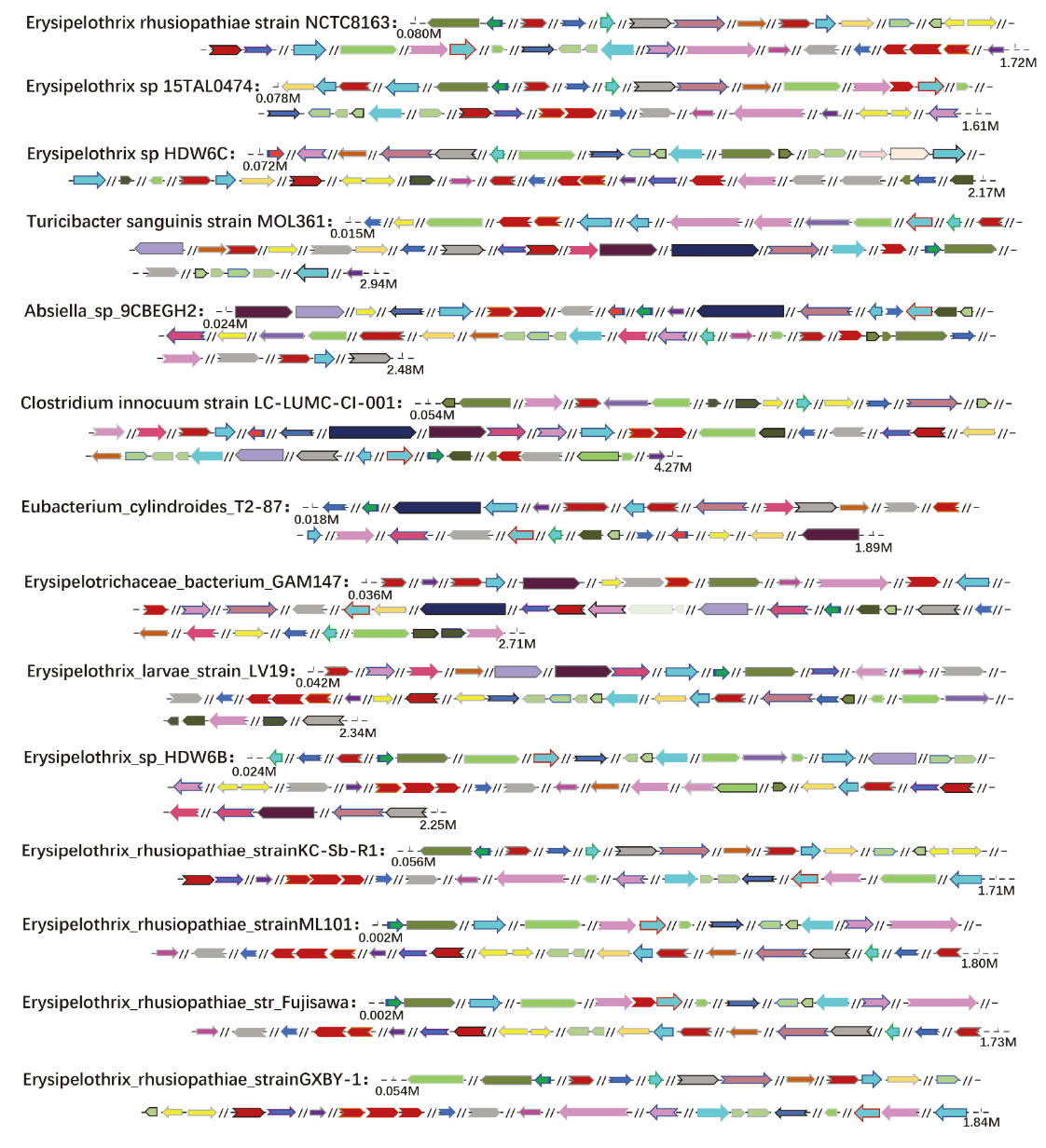


**Supplementary Figure 7.** The organization of the genes related to the metabolisms of 13 carbohydrate substrates in the genomes of 14 Erysipelotrichaceae strains downloaded from the NCBI database. Each colored arrow represents a gene involving in the metabolism of carbohydrate substrates. The detailed information about genes represented by each arrows is displayed in Table S6.
