# Supplementary material for: Isolation and genomic characterization of five novel strains of Erysipelotrichaceae from commercial pigs
Source: BMC Microbiol. 2021 Apr 23;21:125. doi: 10.1186/s12866-021-02193-3 (PMC8063399; doi:10.1186/s12866-021-02193-3)
Supplement: Supplementary file 9 — Additional file 9: Table S2. Statistic description for sequencing data of five isolates. [file 12866_2021_2193_MOESM9_ESM.docx]

| **Table S2. Statistic description for sequencing data of five isolates.** | | | |  |  |  |  |  |  |  |
| --- | --- | --- | --- | --- | --- | --- | --- | --- | --- | --- |
|  |  |  |  |  |  |  |  |  |  |  |
| **Type** | **4-8-110** | | **4-15-1** | | **4-2-123** | | **4-6-57** | | **5-26-39** | |
|  | **Raw Reads** | **Filtered Reads** | **Raw Reads** | **Filtered Reads** | **Raw Reads** | **Filtered Reads** | **Raw Reads** | **Filtered Reads** | **Raw Reads** | **Filtered Reads** |
| Bases(bp) | 2,002,277,182 | 1,927,595,658 | 2,201,402,213 | 2,106,256,745 | 2,394,840,742 | 2,233,509,214 | 1,408,101,386 | 1,344,056,230 | 2,384,939,891 | 2,236,819,919 |
| Reads number | 83,501 | 78,325 | 97,313 | 90,552 | 119,633 | 105,007 | 72,064 | 64,378 | 119,430 | 105,677 |
| Reads mean length (bp) | 23,979.08 | 24,610.22 | 22,621.87 | 23,260.19 | 20,018.23 | 21,270.10 | 19,539.60 | 20,877.57 | 19,969.35 | 21,166.57 |
| Reads N50 (bp) | 35,389 | 35,426 | 33,654 | 33,740 | 30,084 | 30,057 | 28,894 | 28,884 | 28,739 | 28,733 |
| Longest Reads (bp) | 194,387 | 194,387 | 187,828 | 187,828 | 178,336 | 178,336 | 139,114 | 139,114 | 151,717 | 151,717 |
| mean quality | 9.53 | 9.67 | 9.45 | 9.61 | 9.33 | 9.58 | 9.74 | 9.93 | 9.31 | 9.52 |
| # A total of 48,002 reads with 0.54 Gbases were excluded because of low quality (with mean qscore template <7) and short reads (length < 1,000). | | | | | | | | |  |  |
