# Supplementary material for: Isolation and genomic characterization of five novel strains of Erysipelotrichaceae from commercial pigs
Source: BMC Microbiol. 2021 Apr 23;21:125. doi: 10.1186/s12866-021-02193-3 (PMC8063399; doi:10.1186/s12866-021-02193-3)
Supplement: Supplementary file 10 — Additional file 10: Table S3. Genome size, the number of contigs and sequencing depth for each strain. [file 12866_2021_2193_MOESM10_ESM.docx]

| **Table S3. Genome size, the number of contigs and sequencing depth for each strain.** | | | |  |  |
| --- | --- | --- | --- | --- | --- |
|  |  |  |  |  |  |
| **Type** | **4-8-110** | **4-15-1** | **4-2-123** | **4-6-57** | **5-26-39** |
| Estimate strain's name | Erysipelotrichaceae bacterium OH741_COT-311-4-8-110 | Erysipelotrichaceae bacterium OH741_COT-311-4-15-1 | Eubacterium sp. AM28-29-4-2-123 | Faecalitalea cylindroides-4-6-57 | Faecalitalea cylindroides-5-26-39 |
| Genome size (bp) | 2,329,541 | 2,417,034 | 2,453,470 | 2,344,803 | 2,279,876 |
| Contig Number | 1 | 2 | 1 | 1 | 1 |
| Contig N50 (bp) | 2,329,541 | 2,352,596 | 2,453,470 | 2,344,803 | 2,279,876 |
| Longest contig (bp) | 2,329,541 | 2,352,596 | 2,453,470 | 2,344,803 | 2,279,876 |
| Shortest contig (bp) | - | 64,438 | - | - | - |
| Sequencing depth(X) | 790.26 | 845.52/256.40 | 839.57 | 547.47 | 921.43 |
