# Supplementary material for: Isolation and genomic characterization of five novel strains of Erysipelotrichaceae from commercial pigs
Source: BMC Microbiol. 2021 Apr 23;21:125. doi: 10.1186/s12866-021-02193-3 (PMC8063399; doi:10.1186/s12866-021-02193-3)
Supplement: Supplementary file 11 — Additional file 11: Table S4. The ANI values between the five strains based on whole genomes. [file 12866_2021_2193_MOESM11_ESM.docx]

| **Table S4.The ANI values among whole genome sequences of five isolated strains.** | | | | | | | | |
| --- | --- | --- | --- | --- | --- | --- | --- | --- |
|  |  |  |  |  |  |  |  |  |
|  | 4-2-123 | 4-6-57 | 4-8-110 | 4-15-1 | 5-26-39 |  |  |  |
| 4-2-123 | 100 |  |  |  |  |  |  |  |
| 4-6-57 | 72.87 | 100 |  |  |  |  |  |  |
| 4-8-110 | 67.83 | 68 | 100 |  |  |  |  |  |
| 4-15-1 | 67.99 | 72.72 | 97.35 | 100 |  |  |  |  |
| 5-26-39 | 72.26 | 98.87 | 66.77 | 70.84 | 100 |  |  |  |
|  |  |  |  |  |  |  |  |  |
