# Supplementary material for: Isolation and genomic characterization of five novel strains of Erysipelotrichaceae from commercial pigs
Source: BMC Microbiol. 2021 Apr 23;21:125. doi: 10.1186/s12866-021-02193-3 (PMC8063399; doi:10.1186/s12866-021-02193-3)
Supplement: Supplementary file 12 — Additional file 12: Table S5. Genome structures predicted for five isolates. [file 12866_2021_2193_MOESM12_ESM.docx]

| **Table S5. Genome structures predicted for five isolates.** | | | | |  |  |  |  |  |  |  |
| --- | --- | --- | --- | --- | --- | --- | --- | --- | --- | --- | --- |
|  |  |  |  |  |  |  |  |  |  |  |  |
| **Type** | | **4-8-110** | | **4-15-1** | | **4-2-123** | | **4-6-57** | | **5-26-39** | |
|  |  | **Number** | **Length (bp)** | **Number** | **Length (bp)** | **Number** | **Length (bp)** | **Number** | **Length (bp)** | **Number** | **Length (bp)** |
| tRNA |  | 73 | 5709 | 73 | 5709 | 53 | 4148 | 49 | 3841 | 48 | 3766 |
| rRNA | 16S | 4 | 6094 | 4 | 6101 | 7 | 10705 | 5 | 7576 | 5 | 7563 |
| rRNA | 23S | 4 | 11570 | 4 | 11569 | 7 | 20279 | 5 | 14455 | 5 | 14450 |
| rRNA | 5S | 4 | 452 | 4 | 452 | 7 | 791 | 5 | 565 | 5 | 565 |
| CDS |  | 2281 | 2095929 | 2398 | 2173398 | 2475 | 2155674 | 2339 | 2107584 | 2286 | 2048133 |
| CRISPR |  | 1 | 235 | 0 | 0 | 0 | 0 | 3 | 4985 | 1 | 1951 |
| genomic_island |  | 0 | 0 | 0 | 0 | 0 | 0 | 0 | 0 | 0 | 0 |
