# Supplementary material for: Isolation and genomic characterization of five novel strains of Erysipelotrichaceae from commercial pigs
Source: BMC Microbiol. 2021 Apr 23;21:125. doi: 10.1186/s12866-021-02193-3 (PMC8063399; doi:10.1186/s12866-021-02193-3)
Supplement: Supplementary file 13 — Additional file 13: Table S6. Annotation of proteins shared by five isolates with KEGG pathways at the different levels. [file 12866_2021_2193_MOESM13_ESM.docx]

| **Table S6. Annotation of proteins shared by five isolates with KEGG pathways at the different levels.** | | | |  |  |  |  |  |  |  |  |  |  |  |  |  |  |  |  |  |  |  |  |  |  |  |  |  |  |  |  |  |  |  |  |  |  |
| --- | --- | --- | --- | --- | --- | --- | --- | --- | --- | --- | --- | --- | --- | --- | --- | --- | --- | --- | --- | --- | --- | --- | --- | --- | --- | --- | --- | --- | --- | --- | --- | --- | --- | --- | --- | --- | --- |
|  |  |  |  |  |  |  |  |  |  |  |  |  |  |  |  |  |  |  |  |  |  |  |  |  |  |  |  |  |  |  |  |  |  |  |  |  |  |
| **Function term at the level 1** | **Function term at the level 2** | **Function term at the level 3** | **Percentage of the proteins involved in the pathway in all 67 shared proteins** | **Proteins involving in the pathway** |  |  |  |  |  |  |  |  |  |  |  |  |  |  |  |  |  |  |  |  |  |  |  |  |  |  |  |  |  |  |  |  |  |
| Metabolism | Amino acid metabolism | Histidine metabolism | 1.49 | euc: EC1_15900 |  |  |  |  |  |  |  |  |  |  |  |  |  |  |  |  |  |  |  |  |  |  |  |  |  |  |  |  |  |  |  |  |  |
| Metabolism | Amino acid metabolism | D-Glutamine and D-glutamate metabolism | 1.49 | euc: EC1_02350 |  |  |  |  |  |  |  |  |  |  |  |  |  |  |  |  |  |  |  |  |  |  |  |  |  |  |  |  |  |  |  |  |  |
| Metabolism | Amino acid metabolism | Glutathione metabolism | 1.49 | erb: A4V01_00545 |  |  |  |  |  |  |  |  |  |  |  |  |  |  |  |  |  |  |  |  |  |  |  |  |  |  |  |  |  |  |  |  |  |
| Metabolism | Biosynthesis of other secondary metabolites | Streptomycin biosynthesis | 2.99 | euc: EC1_19810, fro: AALO17_14070 |  |  |  |  |  |  |  |  |  |  |  |  |  |  |  |  |  |  |  |  |  |  |  |  |  |  |  |  |  |  |  |  |  |
| Metabolism | Carbohydrate metabolism | Pentose and glucuronate interconversions | 1.49 | euc: EC1_02390 |  |  |  |  |  |  |  |  |  |  |  |  |  |  |  |  |  |  |  |  |  |  |  |  |  |  |  |  |  |  |  |  |  |
| Metabolism | Carbohydrate metabolism | Fructose and mannose metabolism | 1.49 | euc: EC1_05580 |  |  |  |  |  |  |  |  |  |  |  |  |  |  |  |  |  |  |  |  |  |  |  |  |  |  |  |  |  |  |  |  |  |
| Metabolism | Carbohydrate metabolism | Starch and sucrose metabolism | 1.49 | fro: AALO17_14070 |  |  |  |  |  |  |  |  |  |  |  |  |  |  |  |  |  |  |  |  |  |  |  |  |  |  |  |  |  |  |  |  |  |
| Metabolism | Carbohydrate metabolism | Amino sugar and nucleotide sugar metabolism | 1.49 | fro: AALO17_14070 |  |  |  |  |  |  |  |  |  |  |  |  |  |  |  |  |  |  |  |  |  |  |  |  |  |  |  |  |  |  |  |  |  |
| Metabolism | Carbohydrate metabolism | Glycolysis / Gluconeogenesis | 4.48 | fro: AALO17_07220, euc: EC1_05580, fro: AALO17_14070 |  |  |  |  |  |  |  |  |  |  |  |  |  |  |  |  |  |  |  |  |  |  |  |  |  |  |  |  |  |  |  |  |  |
| Metabolism | Carbohydrate metabolism | Pentose phosphate pathway | 5.97 | euc: EC1_05580, euc: EC1_08690, fro: AALO17_14070, euc: EC1_02390 |  |  |  |  |  |  |  |  |  |  |  |  |  |  |  |  |  |  |  |  |  |  |  |  |  |  |  |  |  |  |  |  |  |
| Metabolism | Carbohydrate metabolism | Galactose metabolism | 5.97 | fro: AALO17_14070, erb: A4V01_20725, erb: A4V01_15655, erb: A4V01_20740 |  |  |  |  |  |  |  |  |  |  |  |  |  |  |  |  |  |  |  |  |  |  |  |  |  |  |  |  |  |  |  |  |  |
| Metabolism | Energy metabolism | Methane metabolism | 1.49 | fro: AALO17_07220 |  |  |  |  |  |  |  |  |  |  |  |  |  |  |  |  |  |  |  |  |  |  |  |  |  |  |  |  |  |  |  |  |  |
| Metabolism | Energy metabolism | Carbon fixation in photosynthetic organisms | 2.99 | euc: EC1_08690, euc: EC1_02390 |  |  |  |  |  |  |  |  |  |  |  |  |  |  |  |  |  |  |  |  |  |  |  |  |  |  |  |  |  |  |  |  |  |
| Metabolism | Glycan biosynthesis and metabolism | Peptidoglycan biosynthesis | 2.99 | euc: EC1_02350, erb: A4V01_15245 |  |  |  |  |  |  |  |  |  |  |  |  |  |  |  |  |  |  |  |  |  |  |  |  |  |  |  |  |  |  |  |  |  |
| Metabolism | Metabolism of cofactors and vitamins | Porphyrin and chlorophyll metabolism | 1.49 | euc: EC1_14990 |  |  |  |  |  |  |  |  |  |  |  |  |  |  |  |  |  |  |  |  |  |  |  |  |  |  |  |  |  |  |  |  |  |
| Metabolism | Metabolism of cofactors and vitamins | Thiamine metabolism | 2.99 | erb: A4V01_12305, euc: EC1_14650 |  |  |  |  |  |  |  |  |  |  |  |  |  |  |  |  |  |  |  |  |  |  |  |  |  |  |  |  |  |  |  |  |  |
| Metabolism | Metabolism of terpenoids and polyketides | Biosynthesis of ansamycins | 1.49 | euc: EC1_08690 |  |  |  |  |  |  |  |  |  |  |  |  |  |  |  |  |  |  |  |  |  |  |  |  |  |  |  |  |  |  |  |  |  |
| Metabolism | Metabolism of terpenoids and polyketides | Terpenoid backbone biosynthesis | 2.99 | erb: A4V01_12305, erb: A4V01_04415 |  |  |  |  |  |  |  |  |  |  |  |  |  |  |  |  |  |  |  |  |  |  |  |  |  |  |  |  |  |  |  |  |  |
| Metabolism | Nucleotide metabolism | Pyrimidine metabolism | 2.99 | erb: A4V01_14355, erb: A4V01_01745 |  |  |  |  |  |  |  |  |  |  |  |  |  |  |  |  |  |  |  |  |  |  |  |  |  |  |  |  |  |  |  |  |  |
| Metabolism | Nucleotide metabolism | Purine metabolism | 5.97 | euc: EC1_21030, fro: AALO17_14070, erb: A4V01_14355, erb: A4V01_01745 |  |  |  |  |  |  |  |  |  |  |  |  |  |  |  |  |  |  |  |  |  |  |  |  |  |  |  |  |  |  |  |  |  |
| Metabolism | Xenobiotics biodegradation and metabolism | Drug metabolism - other enzymes | 1.49 | euc: EC1_21030 |  |  |  |  |  |  |  |  |  |  |  |  |  |  |  |  |  |  |  |  |  |  |  |  |  |  |  |  |  |  |  |  |  |
| Cellular Processes | Cell growth and death | Cell cycle - Caulobacter | 1.49 | erb: A4V01_15245 |  |  |  |  |  |  |  |  |  |  |  |  |  |  |  |  |  |  |  |  |  |  |  |  |  |  |  |  |  |  |  |  |  |
| Cellular Processes | Cellular community - prokaryotes | Quorum sensing | 1.49 | euc: EC1_13960 |  |  |  |  |  |  |  |  |  |  |  |  |  |  |  |  |  |  |  |  |  |  |  |  |  |  |  |  |  |  |  |  |  |
| Environmental Information Processing | Membrane transport | ABC transporters | 1.49 | euc: EC1_06850 |  |  |  |  |  |  |  |  |  |  |  |  |  |  |  |  |  |  |  |  |  |  |  |  |  |  |  |  |  |  |  |  |  |
| Environmental Information Processing | Membrane transport | Bacterial secretion system | 1.49 | euc: EC1_13960 |  |  |  |  |  |  |  |  |  |  |  |  |  |  |  |  |  |  |  |  |  |  |  |  |  |  |  |  |  |  |  |  |  |
| Environmental Information Processing | Membrane transport | Phosphotransferase system (PTS) | 2.99 | erb: A4V01_20740, erb: A4V01_20725 |  |  |  |  |  |  |  |  |  |  |  |  |  |  |  |  |  |  |  |  |  |  |  |  |  |  |  |  |  |  |  |  |  |
| Environmental Information Processing | Signal transduction | HIF-1 signaling pathway | 1.49 | fro: AALO17_07220 |  |  |  |  |  |  |  |  |  |  |  |  |  |  |  |  |  |  |  |  |  |  |  |  |  |  |  |  |  |  |  |  |  |
| Genetic Information Processing | Folding, sorting and degradation | Protein export | 1.49 | euc: EC1_13960 |  |  |  |  |  |  |  |  |  |  |  |  |  |  |  |  |  |  |  |  |  |  |  |  |  |  |  |  |  |  |  |  |  |
| Genetic Information Processing | Folding, sorting and degradation | Sulfur relay system | 1.49 | euc: EC1_14650 |  |  |  |  |  |  |  |  |  |  |  |  |  |  |  |  |  |  |  |  |  |  |  |  |  |  |  |  |  |  |  |  |  |
| Genetic Information Processing | Folding, sorting and degradation | RNA degradation | 2.99 | fro: AALO17_10320, fro: AALO17_07220 |  |  |  |  |  |  |  |  |  |  |  |  |  |  |  |  |  |  |  |  |  |  |  |  |  |  |  |  |  |  |  |  |  |
| Genetic Information Processing | Replication and repair | Nucleotide excision repair | 1.49 | euc: EC1_18710 |  |  |  |  |  |  |  |  |  |  |  |  |  |  |  |  |  |  |  |  |  |  |  |  |  |  |  |  |  |  |  |  |  |
| Genetic Information Processing | Replication and repair | Mismatch repair | 2.99 | erb: A4V01_14355, erb: A4V01_01745 |  |  |  |  |  |  |  |  |  |  |  |  |  |  |  |  |  |  |  |  |  |  |  |  |  |  |  |  |  |  |  |  |  |
| Genetic Information Processing | Replication and repair | DNA replication | 4.48 | erb: A4V01_14355, erb: A4V01_01745, erb: A4V01_12570 |  |  |  |  |  |  |  |  |  |  |  |  |  |  |  |  |  |  |  |  |  |  |  |  |  |  |  |  |  |  |  |  |  |
| Genetic Information Processing | Replication and repair | Homologous recombination | 4.48 | fro: AALO17_13300, erb: A4V01_14355, erb: A4V01_01745 |  |  |  |  |  |  |  |  |  |  |  |  |  |  |  |  |  |  |  |  |  |  |  |  |  |  |  |  |  |  |  |  |  |
| Genetic Information Processing | Translation | Aminoacyl-tRNA biosynthesis | 5.97 | euc: EC1_17240, euc: EC1_14990, euc: EC1_12120, fro: AALO17_12240, |  |  |  |  |  |  |  |  |  |  |  |  |  |  |  |  |  |  |  |  |  |  |  |  |  |  |  |  |  |  |  |  |  |
| Genetic Information Processing | Translation | Ribosome | 7.46 | euc: EC1_03080, euc: EC1_07260, erb: A4V01_21885, euc: EC1_07010, euc: EC1_06820 |  |  |  |  |  |  |  |  |  |  |  |  |  |  |  |  |  |  |  |  |  |  |  |  |  |  |  |  |  |  |  |  |  |
| Human Diseases | Drug resistance: antimicrobial | Vancomycin resistance | 1.49 | erb: A4V01_15245 |  |  |  |  |  |  |  |  |  |  |  |  |  |  |  |  |  |  |  |  |  |  |  |  |  |  |  |  |  |  |  |  |  |
| Human Diseases | Infectious disease: bacterial | Legionellosis | 1.49 | fro: AALO17_10660 |  |  |  |  |  |  |  |  |  |  |  |  |  |  |  |  |  |  |  |  |  |  |  |  |  |  |  |  |  |  |  |  |  |
| Others | Others | Others | 4.48 | fro: AALO17_07220, euc: EC1_08690, euc: EC1_02390 |  |  |  |  |  |  |  |  |  |  |  |  |  |  |  |  |  |  |  |  |  |  |  |  |  |  |  |  |  |  |  |  |  |
| Others | Others | Others | 5.97 | fro: AALO17_07220, euc: EC1_08690, euc: EC1_02390, euc: EC1_15900 |  |  |  |  |  |  |  |  |  |  |  |  |  |  |  |  |  |  |  |  |  |  |  |  |  |  |  |  |  |  |  |  |  |
| Others | Others | Others | 8.96 | fro: AALO17_07220, euc: EC1_05580, euc: EC1_08690, fro: AALO17_14070, euc: EC1_02390, euc: EC1_14990 |  |  |  |  |  |  |  |  |  |  |  |  |  |  |  |  |  |  |  |  |  |  |  |  |  |  |  |  |  |  |  |  |  |
| Others | Others | Others | 11.94 | fro: AALO17_07220, euc: EC1_05580, euc: EC1_19810, euc: EC1_08690, fro: AALO17_14070, euc: EC1_02390, erb: A4V01_12305, erb: A4V01_04415 |  |  |  |  |  |  |  |  |  |  |  |  |  |  |  |  |  |  |  |  |  |  |  |  |  |  |  |  |  |  |  |  |  |
| Others | Others | Others | 13.43 | fro: AALO17_07220, euc: EC1_05580, euc: EC1_08690, fro: AALO17_14070, euc: EC1_02390, euc: EC1_14990, erb: A4V01_12305, euc: EC1_15900, erb: A4V01_04415 |  |  |  |  |  |  |  |  |  |  |  |  |  |  |  |  |  |  |  |  |  |  |  |  |  |  |  |  |  |  |  |  |  |
| Others | Others | Others | 25.37 | erb: A4V01_00545, fro: AALO17_07220, euc: EC1_05580, euc: EC1_21030, euc: EC1_02350, euc: EC1_08690, fro: AALO17_14070, euc: EC1_02390, euc: EC1_14990, erb: A4V01_14355, erb: A4V01_15245, erb: A4V01_12305, erb: A4V01_01745, euc: EC1_15900, euc: EC1_14650, erb: A4V01_15655, erb: A4V01_04415 |  |  |  |  |  |  |  |  |  |  |  |  |  |  |  |  |  |  |  |  |  |  |  |  |  |  |  |  |  |  |  |  |  |
