# Supplementary material for: Isolation and genomic characterization of five novel strains of Erysipelotrichaceae from commercial pigs
Source: BMC Microbiol. 2021 Apr 23;21:125. doi: 10.1186/s12866-021-02193-3 (PMC8063399; doi:10.1186/s12866-021-02193-3)
Supplement: Supplementary file 14 — Additional file 14: Table S7. KEGG orthologues involving the transport (PTS system), catalyzation and regulation of the metabolisms of 13 carbohydrate substrates. [file 12866_2021_2193_MOESM14_ESM.docx]

| **Table S7. KEGG orthologues involving the transport (PTS system), catalyzation and regulation of the metabolisms of 13 carbohydrate substrates.** | | | | | |
| --- | --- | --- | --- | --- | --- |
|  |  |  |  |  |  |
| **Function** | | **K_number** | **K_name** | **Describe** | **legend** |
| Transport | PTS system | K02768 | PTS-Fru-EIIA, fruB | PTS system, fructose-specific IIA component |  |
|  |  | K02769 | PTS-Fru-EIIB, fruA | PTS system, fructose-specific IIB component |  |
|  |  | K02770 | PTS-Fru-EIIC, fruA | PTS system, fructose-specific IIC component | \|  \| \| --- \| |
|  |  | K02744 | PTS-Aga-EIIA, agaF | PTS system, N-acetylgalactosamine-specific IIA component |  |
|  |  | K02745 | PTS-Aga-EIIB, agaV | PTS system, N-acetylgalactosamine-specific IIB component |  |
|  |  | K02746 | PTS-Aga-EIIC, agaW | PTS system, N-acetylgalactosamine-specific IIC component |  |
|  |  | K02747 | PTS-Aga-EIID, agaE | PTS system, N-acetylgalactosamine-specific IID component |  |
|  |  | K02793 | PTS-Man-EIIA, manX | PTS system, mannose-specific IIA component |  |
|  |  | K02794 | PTS-Man-EIIB, manX | PTS system, mannose-specific IIB component |  |
|  |  | K02795 | PTS-Man-EIIC, manY | PTS system, mannose-specific IIC component |  |
|  |  | K02796 | PTS-Man-EIID, manZ | PTS system, mannose-specific IID component |  |
|  |  | K02786 | PTS-Lac-EIIA, lacF | PTS system, lactose-specific IIA component |  |
|  |  | K02788 | PTS-Lac-EIIC, lacE | PTS system, lactose-specific IIC component |  |
|  |  | K02777 | PTS-Glc-EIIA, crr | PTS system, sugar-specific IIA component |  |
|  |  | K02791 | PTS-MalGlc-EIIC, malX | PTS system, maltose/glucose-specific IIC component |  |
|  |  | K20118 | PTS-Glc1-EIIC, ptsG, glcA, glcB | PTS system, glucose-specific IIC component | \|  \| \| --- \| |
|  |  | K02819 | PTS-Tre-EIIC, treB | PTS system, trehalose-specific IIC component |  |
| catalyzation | Aldose | K01785 | galM, GALM | aldose 1-epimerase |  |
|  |  | K01619 | deoC, DERA | deoxyribose-phosphate aldolase |  |
|  |  | K16371 | gatZ-kbaZ | D-tagatose-1,6-bisphosphate aldolase subunit GatZ/KbaZ |  |
|  |  | K01624 | FBA, fbaA | fructose-bisphosphate aldolase, class II |  |
|  | Deaminase | K02564 | nagB, GNPDA | glucosamine-6-phosphate deaminase |  |
|  |  | K01443 | nagA, AMDHD2 | N-acetylglucosamine-6-phosphate deacetylase |  |
|  | Dehydratase | K01686 | uxuA | mannonate dehydratase |  |
|  | Dehydrogenase | K00134 | GAPDH, gapA | glyceraldehyde 3-phosphate dehydrogenase |  |
|  | Enolase | K01689 | ENO, eno | enolase |  |
|  | Epimerase | K01783 | rpe, RPE | ribulose-phosphate 3-epimerase |  |
|  | Etherase | K07106 | murQ | N-acetylmuramic acid 6-phosphate etherase |  |
|  | Hydrolase | K01226 | treC | trehalose-6-phosphate hydrolase |  |
|  | Isomerase | K01810 | GPI, pgi | glucose-6-phosphate isomerase |  |
|  |  | K06859 | pgi1 | glucose-6-phosphate isomerase, archaeal |  |
|  |  | K01812 | uxaC | glucuronate isomerase |  |
|  |  | K01809 | manA, MPI | mannose-6-phosphate isomerase |  |
|  |  | K01808 | rpiB | ribose 5-phosphate isomerase B |  |
|  |  | K02082 | agaS | tagatose-6-phosphate ketose/aldose isomerase |  |
|  |  | K01803 | TPI, tpiA | triosephosphate isomerase (TIM) |  |
|  | Kinase | K00850 | pfkA, PFK | 6-phosphofructokinase 1 |  |
|  |  | K00895 | pfp, PFP | diphosphate-dependent phosphofructokinase |  |
|  |  | K00849 | galK | galactokinase |  |
|  |  | K00845 | glk | glucokinase |  |
|  |  | K00927 | PGK, pgk | phosphoglycerate kinase |  |
|  |  | K00873 | PK, pyk | pyruvate kinase |  |
|  |  | K00948 | PRPS, prsA | ribose-phosphate pyrophosphokinase |  |
|  | Mutase | K15633 | gpmI | 2,3-bisphosphoglycerate-independent phosphoglycerate mutase |  |
|  |  | K01835 | pgm | phosphoglucomutase |  |
|  |  | K01839 | deoB | phosphopentomutase |  |
|  | Oxidoreductase | K03737 | por, nifJ | pyruvate-ferredoxin/flavodoxin oxidoreductase | \|  \| \| --- \| |
|  | Phosphorylase | K00688 | PYG, glgP | glycogen phosphorylase | \|  \| \| --- \| |
|  | Pullulanase | K01200 | PYG, glgP | pullulanase | \|  \| \| --- \| |
|  | Reductase | K00040 | uxuB | fructuronate reductase |  |
|  | Glycosidase | K01220 | E3.2.1.85, lacG | 6-phospho-beta-galactosidase | \|  \| \| --- \| |
|  |  | K01222 | E3.2.1.86A, celF | 6-phospho-beta-glucosidase |  |
|  |  | K01223 | E3.2.1.86B, bglA | 6-phospho-beta-glucosidase |  |
|  |  | K01190 | lacZ | beta-galactosidase |  |
|  |  | K01232 | glvA | maltose-6'-phosphate glucosidase |  |
|  | Transferase | K00705 | malQ | 4-alpha-glucanotransferase | \|  \| \| --- \| |
|  |  | K00975 | glgC | glucose-1-phosphate adenylyltransferase |  |
|  | Transketolase | K00615 | E2.2.1.1, tktA, tktB | transketolase |  |
| Regulation | Repressor | K02081 | agaR | DeoR family transcriptional regulator, aga operon transcriptional repressor |  |
|  |  | K03436 | fruR2, fruR | DeoR family transcriptional regulator, fructose operon transcriptional repressor |  |
|  |  | K02530 | lacR | DeoR family transcriptional regulator, lactose phosphotransferase system repressor |  |
