# Supplementary material for: Isolation and genomic characterization of five novel strains of Erysipelotrichaceae from commercial pigs
Source: BMC Microbiol. 2021 Apr 23;21:125. doi: 10.1186/s12866-021-02193-3 (PMC8063399; doi:10.1186/s12866-021-02193-3)
Supplement: Supplementary file 15 — Additional file 15: Table S8. Prediction of PUL in the genomes of Erysipelotrichaceae strains. [file 12866_2021_2193_MOESM15_ESM.docx]

| **Table S8. Prediction of PUL in the genomes of Erysipelotrichaceae strains.** | | | |  |  |  |  |  |  |  |  |  |  |  |  |  |  |  |  |  |  |  |  |  |  |  |  |  |  |  |  |
| --- | --- | --- | --- | --- | --- | --- | --- | --- | --- | --- | --- | --- | --- | --- | --- | --- | --- | --- | --- | --- | --- | --- | --- | --- | --- | --- | --- | --- | --- | --- | --- |
|  |  |  |  |  |  |  |  |  |  |  |  |  |  |  |  |  |  |  |  |  |  |  |  |  |  |  |  |  |  |  |  |
| **Function** | **Candidate substrates** | **4-8-110** | **4-15-1** | **4-2-123** | **4-6-57** | **5-26-39** | **Erysipelothrix_rhusiopathiae_strain_NCTC8163** | **Erysipelothrix_rhusiopathiae_SY1027** | **Erysipelothrix_sp_15TAL0474** | **Erysipelothrix_sp_HDW6A** | **Erysipelothrix_sp_HDW6C** | **Turicibacter_sanguinis_strainMOL361** | **Turicibacter_sp_H121** | **Absiella_argi_JCM30884** | **Absiella_sp_9CBEGH2** | **Clostridium_innocuum_strainATCC14501** | **Clostridium_innocuum_strainLC-LUMC-CI-001** | **Erysipelotrichaceae_bacterium_I46** | **Eubacterium_cylindroides_T2-87** | **Faecalibaculum_rodentium_strain_Alo17** | **Erysipelotrichaceae_bacterium_SG0102** | **Erysipelotrichaceae_bacterium_GAM147** | **Longibaculum_sp_KGMB06250** | **Erysipelothrix_larvae_strain_LV19** | **Erysipelothrix_sp_HDW6B** | **Erysipelothrix_rhusiopathiae_strainKC-Sb-R1** | **Erysipelothrix_rhusiopathiae_strainML101** | **Erysipelothrix_rhusiopathiae_strainZJ** | **Erysipelothrix_rhusiopathiae_str_Fujisawa** | **Erysipelothrix_rhusiopathiae_strainWH13013** | **Erysipelothrix_rhusiopathiae_strainGXBY-1** |
| **degradation** | **carboxymethylcellulose， xylan， beta-glucan， lichenan** | √ | √ | √ | √ | √ | √ | √ | √ | √ | √ |  |  | √ | √ |  | √ | √ | √ |  | √ |  | √ |  | √ | √ | √ | √ | √ | √ | √ |
|  | **xylan， beta-glucan， lichenan** |  |  |  | √ |  |  |  |  |  |  |  |  |  |  |  |  |  |  |  |  | √ | √ |  |  |  |  |  |  |  |  |
|  | **glycosaminoglycan** | √ | √ |  |  |  | √ | √ | √ |  | √ |  |  |  |  |  |  |  |  |  |  |  |  |  | √ | √ | √ | √ | √ | √ | √ |
|  | **unsaturated hyaluronate disaccharide， chondroitin disaccharide** | √ | √ |  |  |  | √ | √ |  |  | √ |  |  |  |  |  |  |  |  |  |  |  |  |  |  | √ | √ | √ | √ | √ | √ |
|  | **N-glycan** | √ | √ |  |  |  | √ | √ |  |  | √ |  |  |  |  |  |  |  |  |  |  |  |  |  |  | √ | √ | √ | √ | √ | √ |
|  | **pectin** | √ | √ |  |  |  | √ | √ | √ |  | √ |  |  |  |  |  |  |  |  |  |  |  |  |  | √ | √ | √ | √ | √ | √ | √ |
|  | **4-methylumbelliferyl 6-azido-6-deoxy-beta-D-galactoside** |  |  |  |  |  |  |  |  |  |  |  | √ |  |  |  |  |  |  |  |  | √ | √ |  |  |  |  |  |  |  |  |
|  | **galactooligosaccharide** |  |  |  |  |  |  |  |  |  |  |  |  |  |  |  |  |  |  |  |  | √ | √ |  |  |  |  |  |  |  |  |
|  | **cellobiose** |  |  |  |  |  |  |  |  |  |  |  | √ |  |  |  |  |  | √ |  |  | √ | √ |  |  |  |  |  |  |  |  |
|  | **mucin** |  |  |  |  |  |  |  |  |  |  | √ | √ |  |  |  |  |  |  | √ |  |  |  |  |  |  |  |  |  |  |  |
|  | **raffinose** |  |  |  |  |  |  |  |  |  |  | √ |  |  |  |  |  |  |  |  |  |  |  |  |  |  |  |  |  |  |  |
|  | **O-glycan， N-glycan** |  |  |  |  |  |  |  |  |  |  |  |  |  |  |  |  |  | √ |  |  |  |  |  |  |  |  |  |  |  |  |
|  | **kestose** |  |  |  |  |  |  |  |  |  |  |  |  |  |  |  |  |  | √ |  |  |  |  |  |  |  |  |  |  |  |  |
| **biosynthesis** | **capsule polysaccharide** | √ | √ |  |  |  |  |  |  |  |  |  |  | √ |  |  |  |  | √ |  |  | √ |  |  |  |  |  |  |  |  |  |
|  | **O-antigen** |  | √ |  |  |  |  |  |  |  |  |  |  |  |  |  |  |  |  |  |  |  |  |  |  |  |  |  |  |  |  |
|  | **exopolysaccharide** |  | √ |  |  |  |  |  |  |  |  |  |  |  |  |  |  |  |  |  |  |  |  |  |  |  |  |  |  |  |  |
|  | **O-antigen， unknown polysaccharide** |  | √ |  |  |  |  |  |  |  |  |  |  |  |  |  |  |  |  |  |  |  |  |  |  |  |  |  |  |  |  |
|  | **exopolysaccharide， lipopolysaccharide， capsule polysaccharide** |  | √ |  |  |  |  |  |  |  |  |  |  |  |  |  |  |  |  |  |  |  |  |  |  |  |  |  |  |  |  |
|  |  |  |  |  |  |  |  |  |  |  |  |  |  |  |  |  |  |  |  |  |  |  |  |  |  |  |  |  |  |  |  |
| Note: “√” means that presence of PUL on the genomes of the strains. | |  |  |  |  |  |  |  |  |  |  |  |  |  |  |  |  |  |  |  |  |  |  |  |  |  |  |  |  |  |  |
